# Standardized protocols for blood collection and analysis in elasmobranchs: a practical guide for clinicians and researchers

**DOI:** 10.3389/fvets.2025.1754037

**Published:** 2026-01-20

**Authors:** Hugo David, Daniel García-Párraga, Nuno Pereira, Pablo Morón-Elorza

**Affiliations:** 1 Oceanário de Lisboa, Lisboa, Portugal; 2 Fundación Oceanogràfic de la Comunitat Valenciana, Valencia, Spain; 3 Department of Pharmacology and Toxicology, Faculty of Veterinary Medicine, Complutense University of Madrid, Madrid, Spain

**Keywords:** biochemistry, chondrichthyes hematology, protein electrophoresis, ray, reference values, shark, shark research, skate

## Abstract

Blood analysis represents a crucial, yet often underutilized, diagnostic tool in elasmobranch medicine. Its broader application has been limited by the absence of standardized protocols and consistent species-specific reference values. The variability in sampling techniques, analytical methods, and data interpretation presents significant challenges for both clinical practices and cross-institutional research. This manuscript offers a comprehensive, evidence-based guideline for blood collection and analysis in elasmobranchs. It details best practices for documenting biological and procedural metadata to enhance sample comparability and provides practical recommendations for venipuncture site selection, blood gas analysis, hematology, biochemistry, and plasma protein electrophoresis. The techniques described are optimized for use with materials and equipment commonly found in veterinary settings. The adoption of these standardized protocols is expected to improve diagnostic accuracy, facilitate the development of reliable species-specific reference ranges, and enable the integration of health data across facilities. Routine implementation in monitored populations is encouraged to set reliable reference physiological levels, support early disease detection and advance veterinary care, as well as contribute to the broader field of elasmobranch health, research and conservation.

## 1 Introduction

Bloodwork is a cornerstone of clinical diagnostics across veterinary species, providing essential insights into health status, disease progression, and physiological responses to environmental or medical interventions. In elasmobranchs, this diagnostic tool is underutilized and poorly standardized. While increasing attention is being given to elasmobranch medicine in recent years, published blood reference values are often based on small, heterogeneous samples, and methodological variability severely limits their applicability across institutions or species (1, 2).

Elasmobranchs, a subclass of cartilaginous fish that includes over 1,200 described species of sharks, rays, and skates, are distributed across all oceans and a variety of freshwater environments (3). They span 13 orders, with members of Carcharhiniforms (ground sharks), Rajiformes (skates), and Myliobatiformes (stingrays) being the most represented in zoological collections and veterinary literature (4, 5).

The results of the blood analysis can be affected by the physiological variability between species, physiological status of the individual, the influence of environmental parameters (e.g., temperature and salinity), and the specifics of sampling technique, such as capture time, restraint method, venipuncture site, anticoagulant used, time from blood collection until sample processing, among others (6). Moreover, variations in equipment sensitivity, sample handling times, and analytical methods can introduce significant inconsistencies, even when samples are obtained under similar conditions (7).

These challenges highlight the need for clear, repeatable guidelines that can be implemented by clinicians and researchers working with elasmobranchs in aquariums, zoos, rehabilitation centers, or field settings. The use of readily available materials, which may be found in any of these facilities, should amplify the compliance of clinicians working with these animals and encourage them to pursue routine blood sampling. This paper presents a practical, experience-based guideline for elasmobranch blood collection and analysis, incorporating both published literature and authors’ experience used successfully in this field. It aims to promote the adoption of harmonized, simplified procedures that improve diagnostic accuracy, support the development of reliable species-specific reference ranges and institutional databases that will ultimately enhance animal welfare and veterinary care, one step closer to providing elasmobranch with the same degree of medical diagnostics as seen in other taxa (8–11).

The protocols and methodologies detailed in this manuscript provide a comprehensive, evidence-based framework for standardizing blood collection and analysis in elasmobranchs. The primary objective of this methodological guide is to enhance diagnostic accuracy and facilitate cross-institutional research comparability by mitigating the significant challenges posed by methodological variability, a well-documented issue in the field.

## 2 Materials and equipment

The successful implementation of this standardized protocol for elasmobranch blood analysis relies on equipment and reagents commonly found in veterinary clinics, zoological institutions, and research laboratories. The following is a comprehensive list of the required materials and equipment, organized by procedural step.

### 2.1 Blood collection

- Syringes: 1–60 mL, depending on animal size and sample volume requirements.
- Needles: 18–23 gauge (G), with length and gauge selected based on species size and venipuncture site (e.g., 23G for small rays, 20G for large sharks).
- Sample tubes: evacuated blood collection tubes or microtainers compatible with the chosen anticoagulant.
  - Anticoagulants: (lithium heparin, sodium heparin, and sodium citrate).
- Permanent Markers for sample labeling.
- Cooler or Insulated Container with ice packs for temporary sample storage and transport.

### 2.2 Blood gas analysis

- Portable Clinical Analyzer: e.g., i-STAT^1^ (Abaxis Inc., Union City, CA, USA) or similar handheld system.
- Single-use cartridges: compatible with the analyzer (e.g., i-STAT CG4+, CG6+, or CG8+; Abaxis Inc., Union City, CA, USA).

### 2.3 Hematology

- Microscope with 10×, 40×, and 100× (oil immersion) objectives.
- Glass microscope slides and coverslips.
- Immersion oil.
- Romanowsky-type stains: e.g., rapid Wright-Giemsa variant as Diff-Quik (Thermo Fisher Scientific, Massachusetts, USA), May-Grünwald-Giemsa, or similar.
- Microhematocrit centrifuge.
- Hematocrit capillary tubes (heparinized and plain).
- Plasma refractometer.
- Improved Neubauer hemocytometer.
- Manual total cell count diluents and stains:
  - Rees-Ecker Fluid.
  - Modified Natt-Herrick’s Solution: *Formulation for elasmobranch osmolarity (~1,000 mOsmol/kg):* Add 0.135 g of sodium chloride (NaCl) and 0.315 g of urea to every 10 mL of standard Natt-Herrick’s solution.

Sample preservation for delayed analysis:

- 10% neutral buffered formalin.
- 1.5 mL microcentrifuge tubes (e.g., Eppendorf tubes).

### 2.4 Plasma biochemistry

- Laboratory centrifuge capable of low-speed spins (e.g., 450–2000 × *g*).
- Laboratory-grade micropipettes and disposable tips for accurate plasma aliquoting and preparation of dilutions.
- Clinical biochemistry analyzer: preferably a system with a wide dynamic range, capable of measuring high urea and electrolyte concentrations. Systems designed for urine electrolyte analysis are often suitable.
- Cryogenic vials for plasma storage.

### 2.5 Plasma protein electrophoresis

- Agarose Gel Electrophoresis System or Capillary Zone Electrophoresis System.

### 2.6 General laboratory and storage

- Vortex Mixer for gentle sample homogenization (optional, samples can be also manually homogenized).
- Timer.
- Freezers:
  −20 °C for short-term plasma storage (up to 3 months).
  −80 °C for long-term plasma archiving.
- Personal Protective Equipment (PPE): lab coat, gloves, and safety glasses.

A detailed overview of the use of the materials described above for standardized blood collection and analysis in elasmobranchs, together with recommended techniques and handling procedures to preserve sample integrity and ensure reliable results, is provided in the following section (Methods). This includes step-by-step guidance on venipuncture sites, needle and syringe selection, anticoagulant use, sample handling, and metadata documentation, establishing a comprehensive framework for consistent and reproducible hematological, biochemical and plasma protein electrophoresis assessments.

## 3 Methods

### 3.1 Blood collection: techniques, handling, and care

The accuracy and clinical value of blood analyses in elasmobranchs depend heavily on proper sampling technique and immediate post-collection handling. Given the unique anatomy and physiology of these species, bloosd collection must be carefully planned to minimize stress, avoid artifact generation, and preserve sample integrity (8).

Prolonged capture efforts can induce stress-related changes in blood chemistry and blood gas analysis, including metabolic acidosis, and compromise the animal’s health significantly. It is critical to limit handling time and physical exertion prior to sampling. Specific care should be taken in gravid female or with those under intense follicular development, were improper restraint, such as too much pressure in the caudal celom or abrupt rotation, can lead to ovarian injury. Additionally, the use of anesthetics and recent feeding status should be considered, as both can alter blood parameters and should be consistently recorded (12–14).

**Figure 1:**
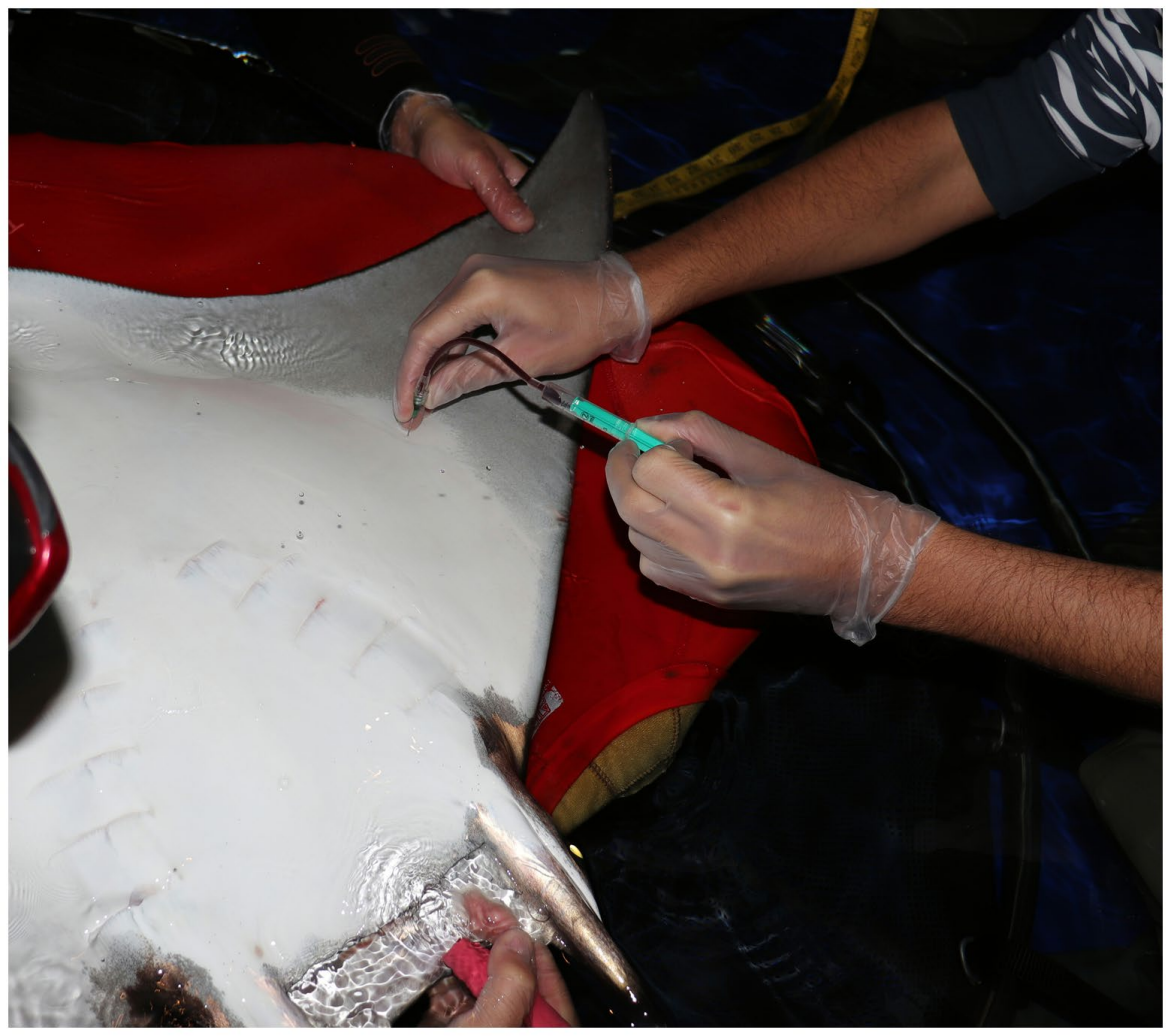
A common eagle ray (*Myliobatis aquila*) placed in dorsal recumbency for blood collection from a wing vein.

**Figure 2:**
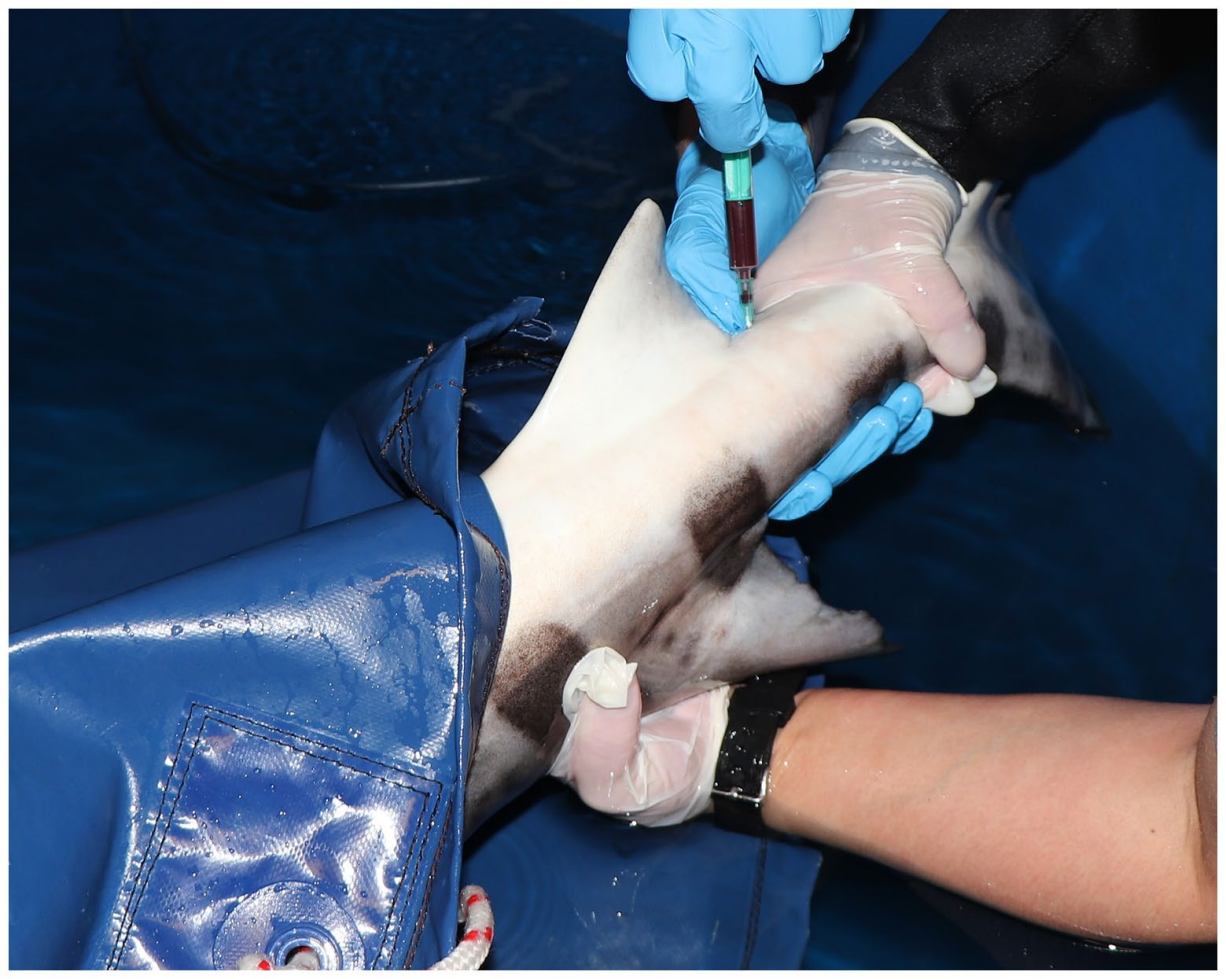
Blood collection in a nursehound shark (*Scyliorhinus stellaris*) showing the ventral approach to the caudal vein.

The choice of venipuncture site varies between rays and sharks and should consider both species-specific anatomy and accessibility. In rays, commonly accessed sites include the wing veins between the ceratotrichia of the pectoral fins (Figure 1) and the caudal vein, which is located ventral to the vertebral column and covered by a cartilage layer that can obstruct needle entry. The access to the mesopterygeal vein has also been described in these animals (15). In sharks, the caudal vein is typically accessed via a ventral midline approach just caudal to the anal fin (Figure 2). A lateral approach may facilitate venipuncture and reduce tissue trauma in species with laterally compressed tails such as *Stegostoma tigrinum* or *Scyliorhinus stellaris* (16). Dorsal sinuses and lateral caudal access may also be appropriate in large-bodied sharks such as *Carcharias taurus* (17). Regardless of the site, consistency in venipuncture location is vital, as analyte values may vary between sampling points (1). For further visual support on venipuncture sites and accesses please refer to the Supplementary figures of this manuscript.

Needle and syringe size should be adapted to the size of the animal. For example, a 23G needle may be appropriate for a 3 kg ray, while a 20G needle may be more suitable for sharks exceeding 20 kg. Depending on the species, needles and syringes should be lightly heparinized, by drawing up and expelling sodium heparin, to minimize clot formation. However, clinicians should be aware that sodium heparin can interfere with electrolyte readings, particularly sodium concentrations. If accurate electrolyte measurements are required, samples should be drawn without anticoagulants and promptly transferred to lithium heparin tubes (18).

Blood volume should not exceed 1% of an animal’s body mass within a 48-h period to avoid compromising health (19). This guideline applies broadly across fish species and is suitable for clinical and routine sampling in elasmobranchs.

Comprehensive metadata accompanying each sample enhances interpretation and comparability. These details should include species, sex, weight, health/physiological status (e.g., pregnancy, post-delivery, neonate, etc.), fasting time, anesthetic use, reason for sampling, venipuncture site, anticoagulant used, diluting solution for total cell counts and biochemistry equipment. Proper record-keeping is particularly important for institutions aiming to build longitudinal databases, since these details can significantly contribute to different results (20–22).

After collection, the first drops of blood should be discarded, when possible, to avoid contamination. Blood should be gently introduced into anticoagulant tubes and mixed by slow inversion for at least 1 min. Tube fill volume should follow manufacturer guidelines, underfilling or overfilling may alter anticoagulant ratios and affect results. The authors use lithium heparin vials as the primary anticoagulant, which produces no apparent artifacts when samples are processed shortly after collection, while citrate is also commonly employed for hematological analyses in elasmobranchs (1).

When analysis is delayed, blood intended for biochemistry and protein electrophoresis should be refrigerated and centrifuged as soon as possible. Plasma should be stored at −20 °C or at −80 °C for long-term storage, maintaining a strict cold chain to minimize degradation. Plasma banking is encouraged whenever feasible to allow for retrospective or expanded testing (23).

Serum is obtained by collecting blood in tubes without anticoagulant, allowing it to clot, and then centrifuging to separate the clotted cellular material. Plasma is collected using anticoagulants and centrifuged before clotting occurs. For most biochemical and protein electrophoretic analyses in elasmobranchs, plasma is preferred due to faster processing and to avoid analyte consumption during clotting (12, 24). Certain assays may require serum to avoid anticoagulant interference (17, 25). The choice of sample matrix should be guided by the specific analytical requirements and validated protocols.

### 3.2 Blood gas analysis

Blood gas analysis should be prioritized immediately after blood collection to ensure reliable results, as gas exchange continues *in vitro* and can quickly alter critical values. Ideally blood should be transferred as soon as possible into the gas analyzer, if feasible directly from the syringe after discarding the first drops. This type of analysis is essential for evaluating respiratory and metabolic status, particularly in the context of anesthesia, transportation, or prolonged restraint.

Key analytes typically include pH, partial pressures of carbon dioxide (pCO₂) and oxygen (pO₂), bicarbonate (HCO₃^−^), base excess (BE) and lactate. These measurements provide insight into acid–base balance, oxygenation status, and anaerobic metabolism, and they can reveal evidence of respiratory compromise or metabolic disturbances such as lactic acidosis (26).

If using a portable analyzer such as the i-STAT (Abaxis Inc., Union City, CA), optimal accuracy is achieved by calibrating the device to the temperature of the animal or its surrounding water (27). These equipment’s are by default assuming a patient temperature of 36 °C as they are built for human medicine, much different from elasmobranch body temperature. Strictly adhere to the manufacturer’s instructions, as even minor errors in cartridge loading can result in analysis failure and loss of data. Alterations in pH (e.g., lower than 7.2) and elevated lactate concentrations (e.g., higher than 5.0 mmol/L) have been associated with stress-induced anaerobic metabolism in elasmobranchs (28, 29).

### 3.3 Hematology

Hematological parameters provide critical insights into a wide array of clinical conditions, including infections, inflammatory diseases, cytotoxic exposures, and environmental stressors. These analyses are relatively low-cost and can be performed in-house (2, 8). Whenever possible, it is recommended that hematological assessments be conducted by the same individual, as proficiency improves with repeated familiarity with species-specific cellular characteristics and it allows for a better comparison of results, as slight differences between operators may occur (30).

Immediately following blood gas analysis, a small drop of blood should be placed directly from the syringe onto a slide to prepare a smear. This should be air-dry completely before fixation and staining. It is also recommended to prepare an additional smear using homogenized blood from the anticoagulant tube, as this often produces clearer smears according to the authors’ experience. Common staining protocols include a rapid Wright-Giemsa such as Diff-Quick (Thermo Fisher Scientific, Massachusetts, USA), Giemsa, and May Grunwald (1). Of these, the rapid Wright-Giemsa is widely available, easy to use, and efficient, making it the preferred option in many clinical environments (31–33).

Leukocytes in elasmobranchs include lymphocytes, monocytes, neutrophils, basophils, and eosinophilic granulocytes, with most cell types sharing general characteristics with those of mammals. Eosinophilic granulocytes can be further divided into fine eosinophilic granulocytes or granulocyte 3 (G3) following Mainwaring and Rowley (34) classification, the equivalent of heterophiles in reptile and avian species, and coarse eosinophilic granulocytes, or G1, the equivalent to eosinophiles in other species. In rays, lymphocytes typically dominate the WBC population, while in sharks, granulocytes can equal or exceed lymphocyte numbers. Neutrophils, or G2, are common in sharks but infrequent in rays and skates. Basophils are rarely observed in sharks but routinely seen in low numbers in rays. In some shark species another peculiar cell may also be found, granulated thrombocytes, or G4. The function of this cell is not totally known yet but is usually counted together with leukocytes (1, 31, 34).

Differentials should be performed using immersion oil (100x) microscopy by counting a minimum of 100 WBCs (ideally 200) and calculating the percentages for each type. These percentages are applied to total WBC counts to derive absolute values. The blood smear should be examined in the central region, where a monolayer of cells is present, avoiding areas with overlapping or excessively deformed cells typically found at the edges. Immature granulocytes should be quantified separately to assess for a left shift, a finding that may indicate inflammation or infection (11). Typically, immature granulocytes are identified as cells the lack nuclear segmentation and a larger cell size. While immature cells may be normally present in some elasmobranchs, nuclei with overlapped segmentation may be misclassified as immature without careful examination. This effect may be worsened by collection technique or smear quality, leading to misinterpretation of hematological status. A rise in granulocyte percentages is usually seen in any inflammatory or infectious condition. A brief description of each type of cell can be found below.

Packed cell volume (PCV) should be determined using a microhematocrit centrifuge at 1300 *g* (approximately 3,500 rpm with a 10 cm radius rotor) for 6 min. This value helps identify dehydration, anemia, or blood loss, and interspecific differences necessitate species-specific reference ranges. Values as low as 12–15% may be normal in some healthy species (33).

Plasma solids are measured using a refractometer but are not reliable indicators of protein levels in elasmobranchs due to elevated urea and electrolyte concentrations. Increases in solids may reflect hyperglobulinemia or accumulation of non-protein solutes like glucose or creatinine. Biochemical protein quantification is preferred over refractometry (35, 36).

Manual counts of erythrocytes, thrombocytes, and leukocytes are typically performed using an improved Neubauer hemocytometer under 40× magnification. The most used diluents for staining and visualization are Rees Ecker Fluid and Natt-Herrick’s solution. When using Rees Ecker Fluid, prepare a 1:100 dilution by mixing 20 μL of blood in 1980 μL of diluent and gently inverting the preparation 10 times to properly homogenize it. Load 10 μL into each side of the chamber. The improved Neubauer chamber has two sides, each with 9 larger squares, with the central one being further divided into 25 squares. Red cells are counted in the central square, specifically in the four in the corner and the one in the middle of the 25 squares and then multiplied by 5,000; leukocytes are counted in four large squares, and the mean value is then multiplied by 1,000. Thrombocytes are counted in all of the central 25 squares, repeated on both sides and then an average of both values is considered. For Natt-Herrick’s solution, use a 1:50 dilution: mix 10 μL of blood with 490 μL of diluent. To match the osmolarity of elasmobranch blood, add 0.135 g of sodium chloride and 0.315 g of urea to every 10 mL of standard Natt-Herrick solution, achieving an osmolality of 1,000 mOsmol/kg. Count cells at 40× magnification (2). Although the Natt-Herricks solution allows easy identification of granulocytes, it does not enable the identification of other leukocytes and thrombocytes. Thus, after a total WBC count in this solution, a proportion should be made with the differential leukocyte count in the smear (ex: Natt-Herricks count = 20, granulocyte count in smear = 50%, total WBC count = 40). Total RBC values are highly variable ranging anywhere from 0.1 to 1.0 × 10^6^ cells/μL. A great majority of the animals present a total WBC count of 10 to 50 × 10^3^ cells/μL.

If hematological analysis cannot be performed immediately, blood smears should still be made at the time of collection. To preserve samples for future total cell counts, the method described by Arnold et al. (37) may be used: mix 50 μL of anticoagulated blood with 200 μL of 10% neutral buffered formalin in a 1.5 mL Eppendorf tube to achieve a 1:5 dilution. Gently invert to mix, avoid hemolysis, and store at room temperature in a dark place. Samples preserved this way may remain viable for up to 30 days. Freezing is not recommended.

Hematological values reported in Table 1 (Results section) demonstrate the diversity of baseline ranges among elasmobranch species, underscoring the need for context-specific interpretation.

**Table 1:** Compilation of hematology results published for different elasmobranch species.

Species (*n*)	*Aetobatus narinari* (17)	*Dasyatis sabina* (10)	*Ginglyostom*a *cirratum* (13)	*Carcharhinus plúmbeu*s (5)	*Carcharhinus melanopterus* (10)	*Cacharhinus* t*aurus* (16)	*Mustelus canis* (20)
STAT	M	M	M	Me	M	M	M
Htc (%)	28	–	24.7	–	28.5	21.1	24
ST (g/dL)	5.7	–	–	–	–	–	–
GR (10^3^/μL)	–	219.0	–	402.0	–	–	–
WBC (10^3^/μL)	18.1	55	–	22.1	14.5	15.7	20.8
L (%)	67.3	69.1	73.3	49.9	49.0	42.0	28.9
FEG (%)	15.4	29.3	24.5	7.0	33.6	37.6	17.7
CEG (%)	10.2			11.0	12.6	10.1	3.1
N (%)	–			6.5	–	–	17.3
B (%)	1.2			–	–	0.3	–
GT (%)	–			21.3	–	–	27.3
M (%)	1.9	1.6	2.2	3.5	4.8	1.7	5.6
L (10^3^/μL)	11.80	–	–	11.00	7.40	12.00	6.13
FEG (10^3^/μL)	2.64	–	–	1.55	5.70	6.80	3.67
CEG (10^3^/μL)	1.76	–	–	2.43	1.83	2.47	0.65
N (10^3^/μL)	–	–	–	1.43	–	–	3.52
B (10^3^/μL)	0.2	–	–	0.00	–	0.05	–
GT (10^3^/μL)	–	–	–	4.70	–	–	5.69
M (10^3^/μL)	0.29	–	–	0.77	0.69	0.39	0.13
REF	(67)	(31)	(31)	(8)	(68)	(55)	(12)

Species (*n*)	*Rhincodon typus* (5)	*Rhincodon typus* (30)	*Rhizoprionodon terraenovae* (30)	*Sphyrna tiburo* (31)	*Squalus acanthias* (30)	*Carchachinus limbatus* (36)
STAT	M	Me	Me	Me	Me	M
Htc (%)	21	20.5	22	25	20	27
ST (g/dL)	–	–	5.5	6.2	4.8	3.2
RBC (10^3^/μL)	–	323.5	–	–	–	0.88
WBC (10^3^/μL)	5.1	9.6	57.2	50.7	40.2	30.9
L (%)	46.0	–	36.0	36.0	35.0	–
FEG (%)	39.5	–	25	20	11.5	–
CEG (%)	6.5	–	11	5	11	–
N (%)	0.9	–	0.0	7.0	17.5	–
B (%)	1.8	–	0.0	0.0	0.0	–
GT (%)	0.0	–	20.5	27.0	18.0	–
M (%)	6.3	–	4	3	5	–
L (10^3^/μL)	2.40	–	19.80	20.50	13.2	–
FEG (10^3^/μL)	2.00	–	16.30	10.60	4.4	–
CEG (10^3^/μL)	0.30	–	6.20	2.20	4.3	–
N (10^3^/μL)	0.10	–	0.00	3.80	6.6	–
B (10^3^/μL)	0.10	–	0.00	0.00	0.0	–
GT (10^3^/μL)	0.00	–	12.60	13.90	6.5	–
M (10^3^/μL)	0.30	–	2.20	1.80	1.7	–
REF	(9)	(21)	(7)	(7)	(7)	(66)

ST, statistic; M, Mean; Me, Median; Hct, hematocrit; TP, total proteins; RBC, red blood cells; WBC, white blood cells; L, lymphocytes; FEG, fine eosinophilic granulocytes; CEG, coarse eosinophilic granulocytes; N, neutrophils; B, basophils; GT, granulated thrombocytes; M, monocytes; Ref, reference. Please note: Granulocytes are combined in certain species as distinctions are not specified in the respective studies.

### 3.4 Peripheral cell identification in a stained blood smear during differential leukocyte counts, a visual guideline

#### 3.4.1 Neutrophils

**Figure 3:**
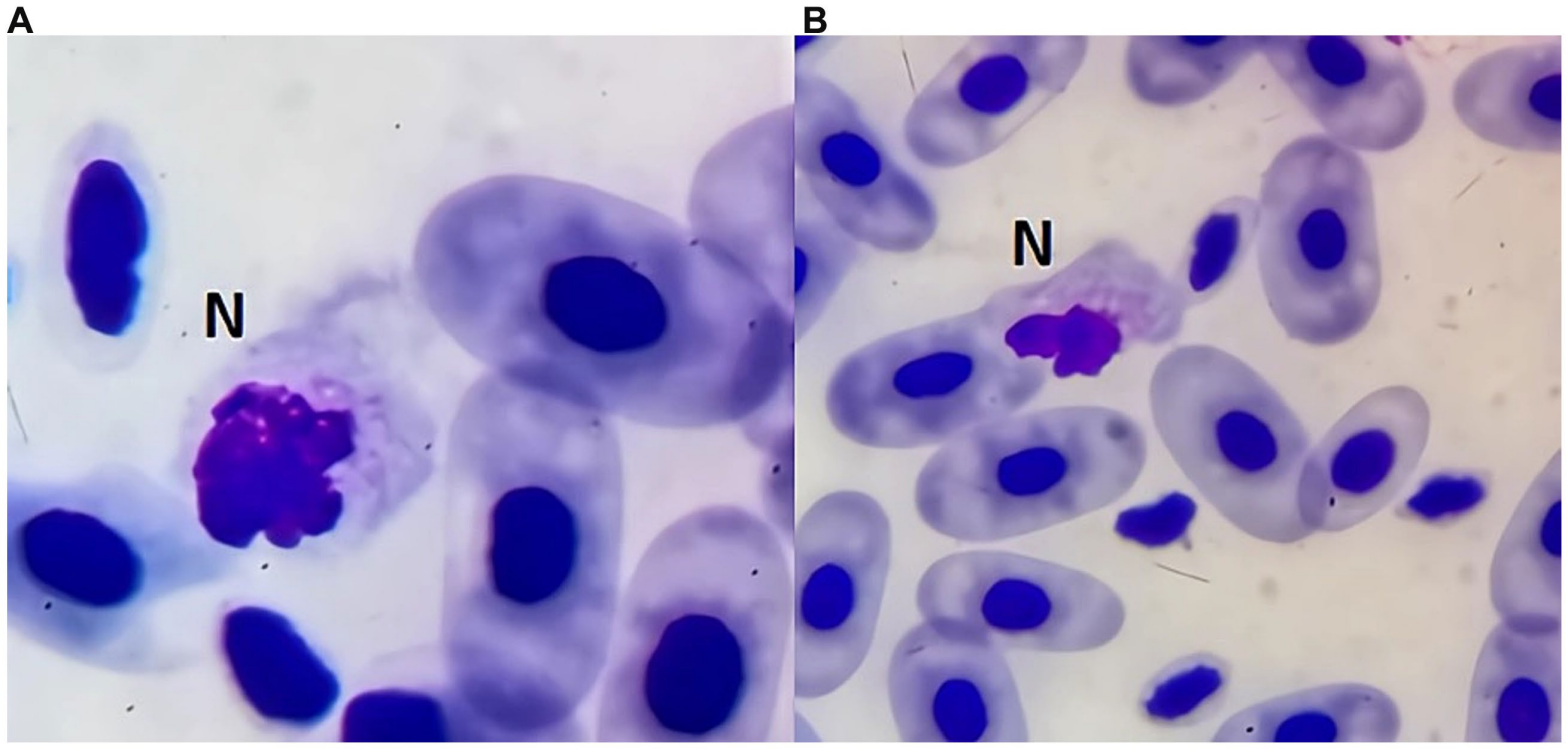
Blood smear of a zebra shark (*Stegostoma tigrinum*), stained with Diff-quick, 100× magnification. Differently shaped neutrophils can be seen in panel **(A,B)**.

Neutrophils, akin to those seen in most mammals, possess a transparent, uncoloured cytoplasm under Giemsa or Diff Quick stains. Their nucleus is irregular, segmented, usually eccentric, and can appear either lobed or non-lobed, with both forms often present within a single blood smear from healthy specimens. Cytoplasm is pale, grey to white, and no granules should be visible. Sometimes stained material appears in the cytoplasm in some samples. Often the cell limits are not well defined. Some cells may have an irregular shape as seen in Figure 3.

#### 3.4.2 Fine eosinophilic granulocytes (FEG)

**Figure 4:**
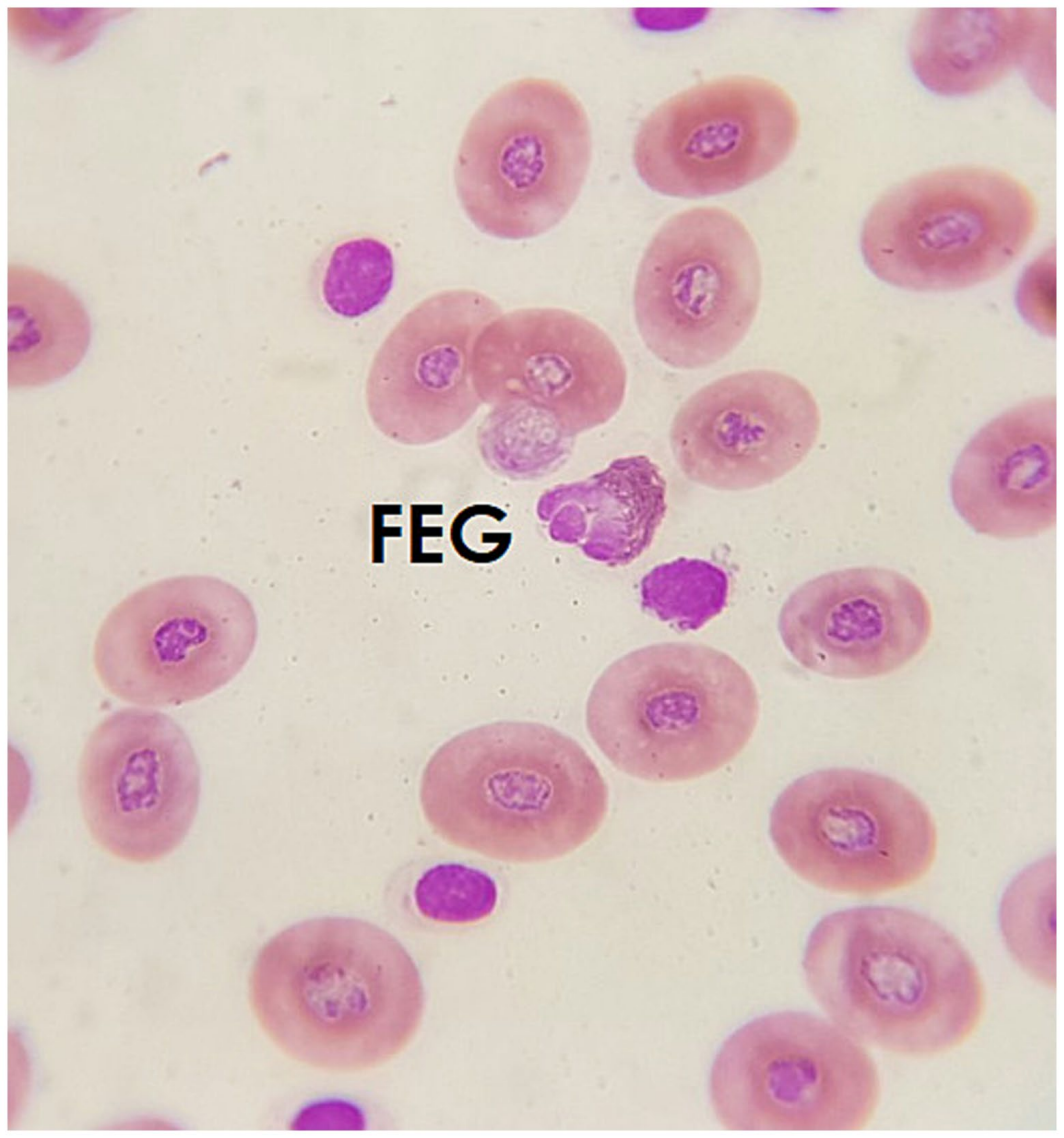
Blood smear of an Atlantic pigmy devil ray (*Mobula hypostoma*), stained with Diff-quick, 100× magnification. A fine eosinophilic granulocyte (FEG) can be seen.

Since the 1980s, various naming systems for eosinophilic cells have been introduced, creating significant confusion, particularly regarding the two types of cells with eosinophilic granules. Cells with cytoplasm filled with elongated, reddish granules, resembling avian heterophils, are often referred to as fine eosinophilic granulocytes (FEG) due to the granule morphology. They present an irregular nucleus, segmented, usually eccentric. The cytoplasm is usually pink, orange, or brown, and granules are regularly observed (Figure 4).

#### 3.4.3 Coarse eosinophilic granulocytes (CEG)

**Figure 5:**
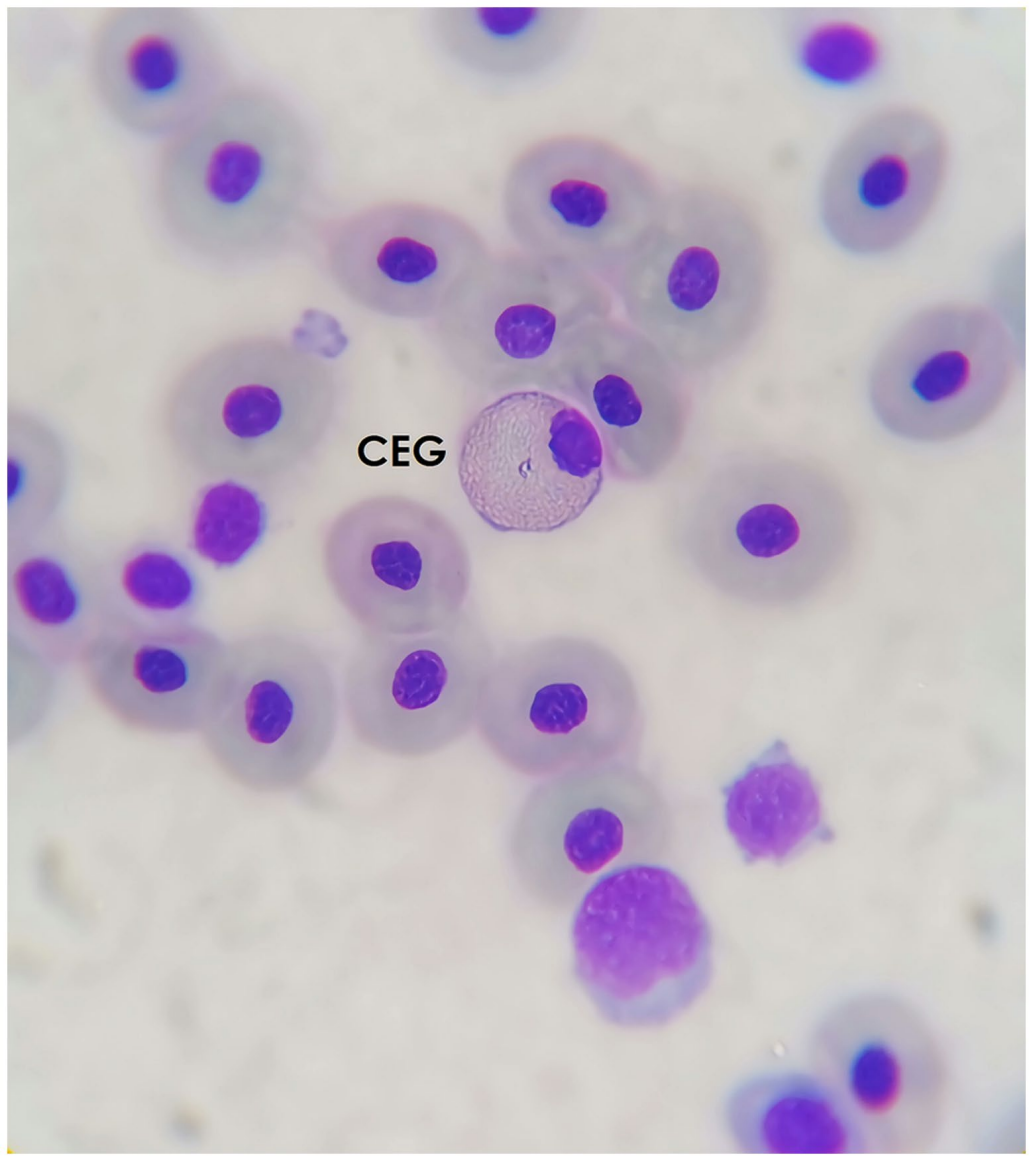
Blood smear of an Atlantic pigmy devil ray (*Mobula hypostoma*), stained with Diff-quick, 100× magnification. A coarse eosinophilic granulocyte (CEG) can be seen.

Cells with oval to round cytoplasmic granules, which frequently exhibit staining properties distinct from heterophils. This cell is regularly found in smaller numbers than the FEG, though this can depend on the species. The main differences from the FEG are the presence of larger and rounded granules with a more intense colour. To the authors’ experience, sometimes the granules are not seen individualized, but rather aggregated, with an bright pink or orange colour, filling the cytoplasm and leaving the nucleus in an excentric position (Figure 5).

#### 3.4.4 Basophils

**Figure 6:**
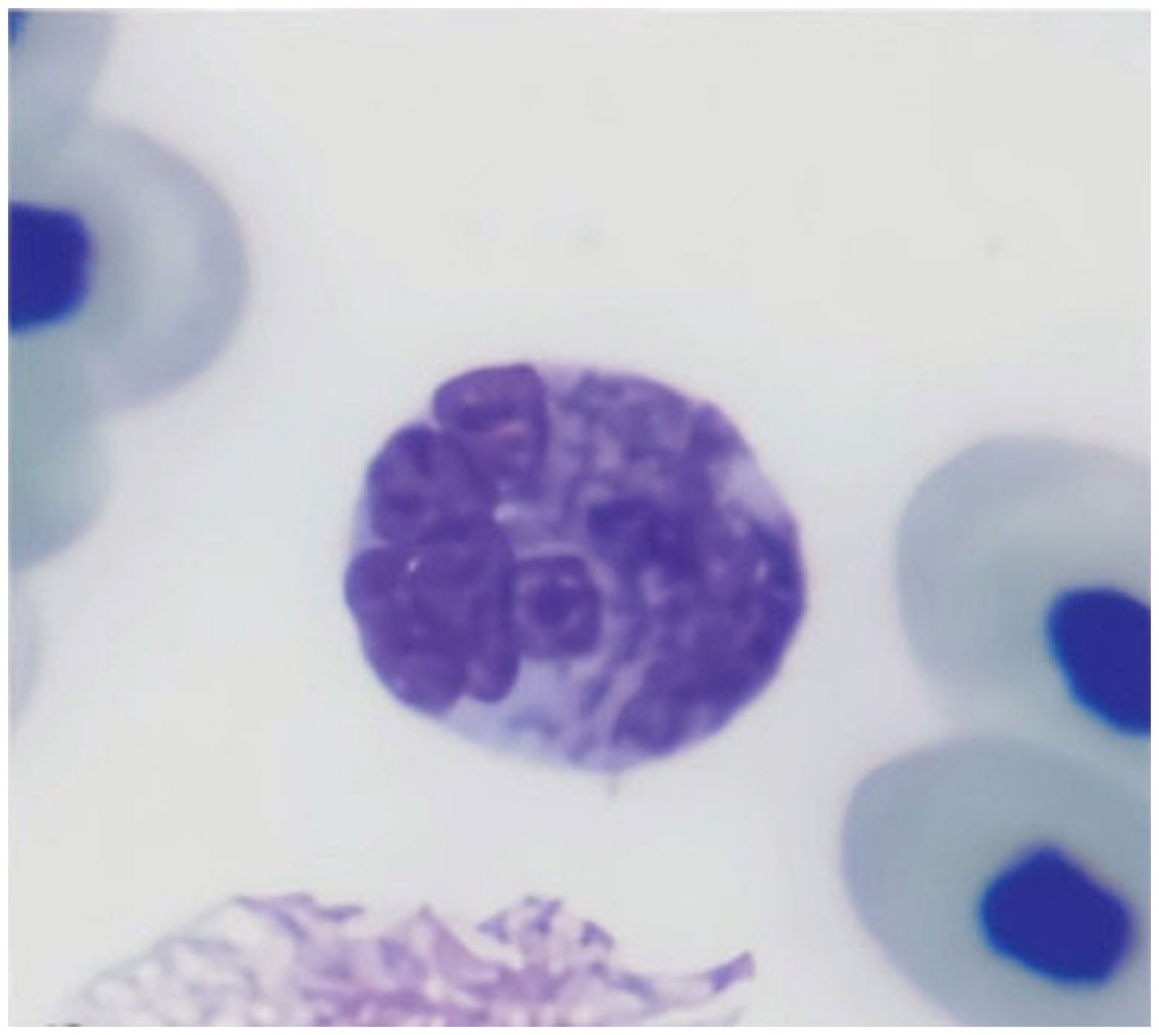
Detail of a basophile (*Aetobatus narinari*), Diff Quick staining. Source: Arnold and Delaune (1).

Basophils are rarely identified. They have a similar presentation to the FEG but with basophilic granules, including granules that overlap the nucleus (Figure 6).

#### 3.4.5 Lymphocytes

**Figure 7:**
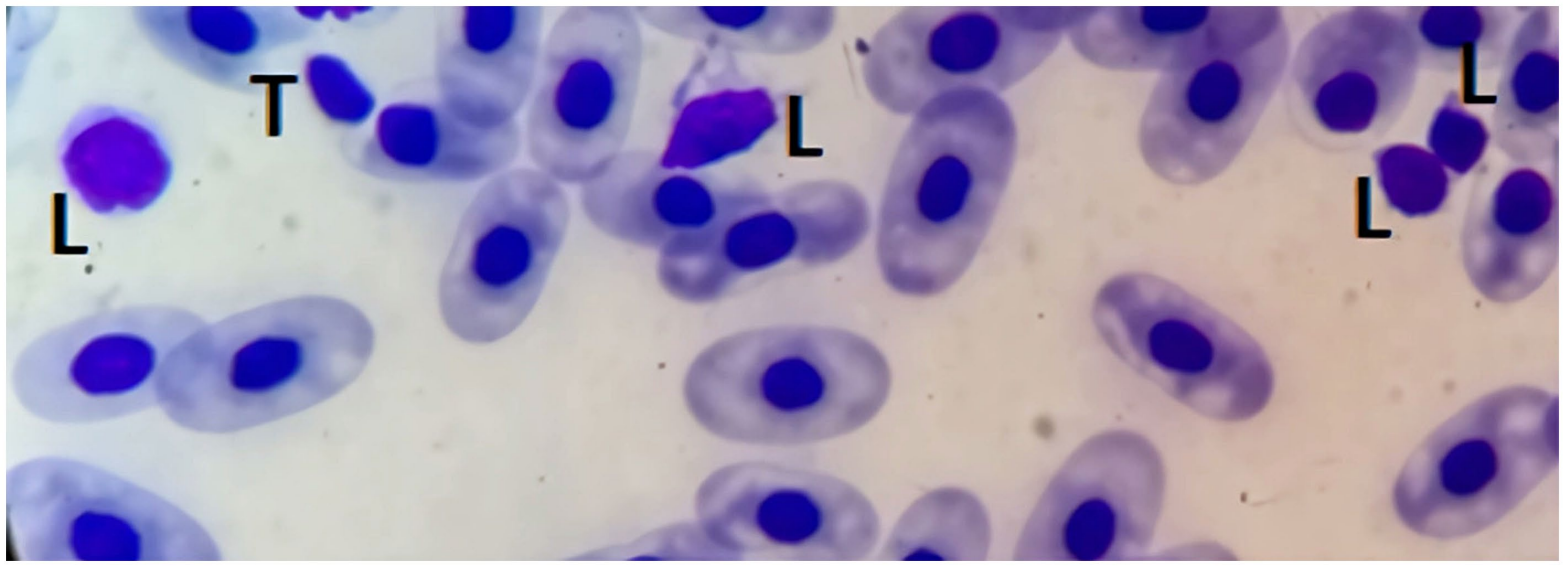
Blood smear of a zebra shark (*Stegostoma tigrinum*), stained with Diff-quick, 100× magnification. Four lymphocytes (L) can be seen. Please note that care should be taken not to mistake lymphocytes with thrombocytes (T).

Lymphocytes are generally small, featuring dense, dark chromatin and a thin rim of pale blue cytoplasm. Occasionally, moderate to large lymphocytes (over 10 μm in size) can be observed in blood smears from healthy sharks. They consist of rounded cells, with a high nucleus-to-cytoplasm ratio. The little cytoplasm that is usually seen is usually blue with a Diff-Quick stain and with slightly irregular limits. Lymphocytes usually show anisocytosis in healthy elasmobranchs, and it is not uncommon for lymphocytes to have an irregular shape (1). Their sometimes-small dimensions and irregular shapes warrant the need for careful observation to distinguished them from thrombocytes (Figure 7).

#### 3.4.6 Monocytes

**Figure 8:**
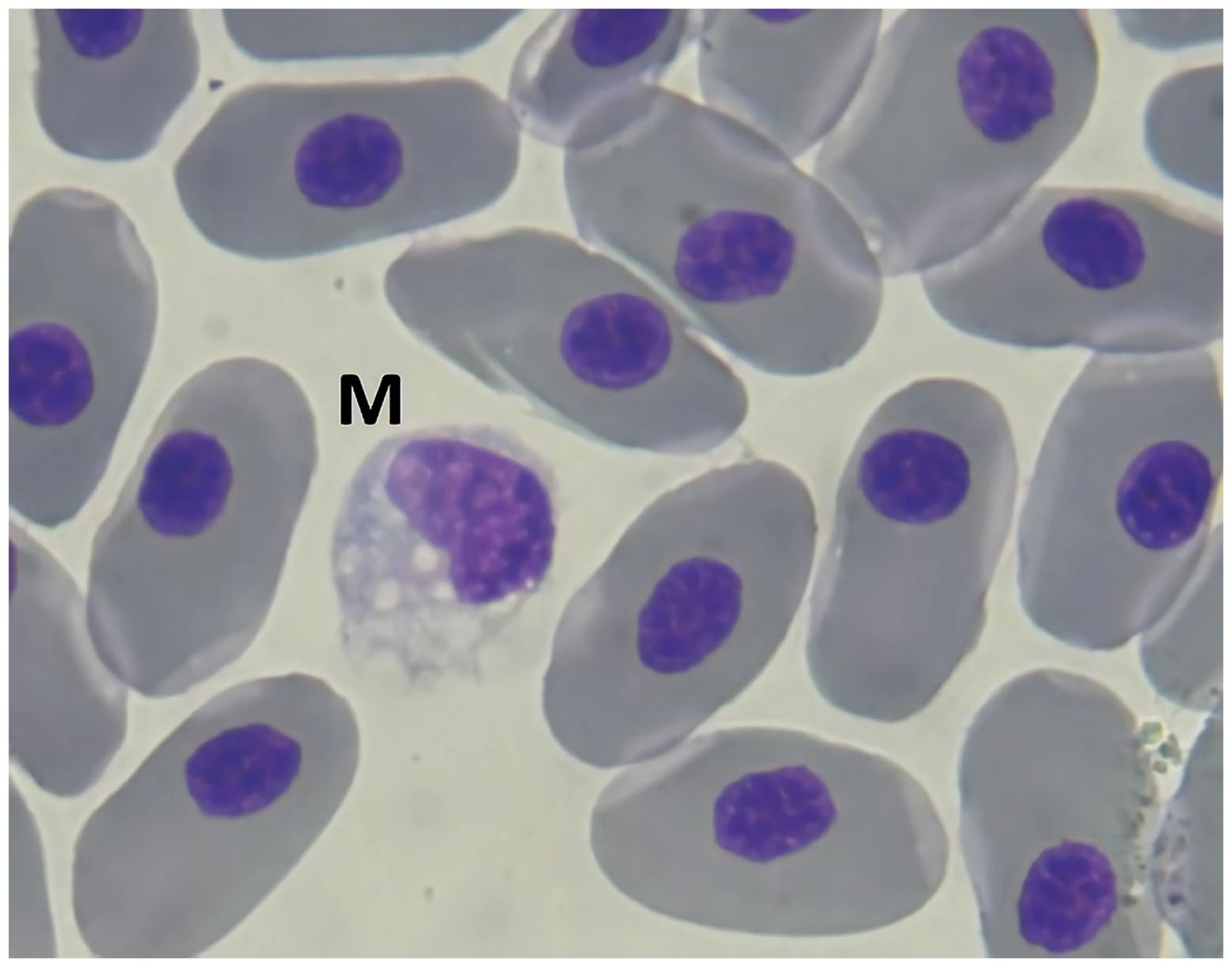
Blood smear of nursehound shark (*Scyliorhinus stellaris*), stained with Diff-quick, 100× magnification. A monocyte (M) with intracytoplasmic vacuoles can be seen.

Monocytes share a similar appearance to their mammalian counterparts (9). They can be distinguished from lymphocytes by their larger volume of blue cytoplasm, which sometimes contain vacuoles. Due to the large size of elasmobranch erythrocytes, the dimensions of monocytes are often overlooked. The nucleus is typically kidney-shaped, although many monocytes may not exhibit this classic feature. The nucleus-to-cytoplasm ratio is generally 1:1, and the cytoplasm ranges from blue to pale in colour (Figure 8).

#### 3.4.7 Thrombocytes

Small cells, with fusiform or elliptical shape and a pale cytoplasm. Thrombocytes may have the same dimensions as smaller lymphocytes, but the fusiform shape and the pale cytoplasm should differentiate them.

**Figure 9:**
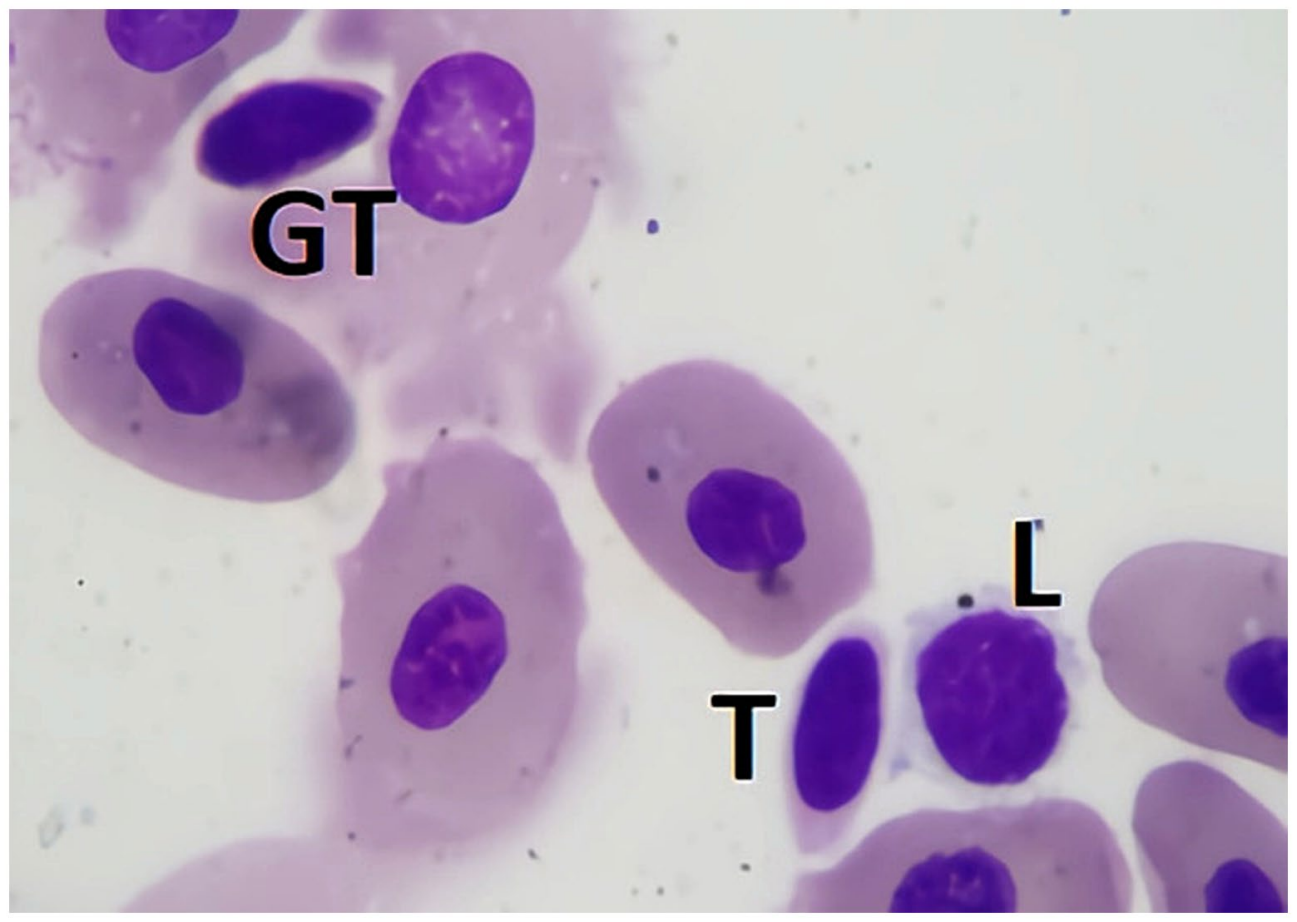
Blood smear of zebra shark (*Stegostoma tigrinum*), stained with Diff-quick, 100× magnification. A thrombocyte (T), a granulated thrombocyte (GT), and a lymphocyte (L) can be seen.

If thrombocytes are seen with cytoplasmic granules, which many times appear as just a rather “red cytoplasm,” these should be counted as Granulated thrombocytes (GT) and be included in the leukocyte differential (Figure 9). This second form of thrombocyte has been observed in certain elasmobranch species, mostly sharks. This cell is similar in size and shape to conventional thrombocytes but is distinguished by cytoplasm densely packed with azurophilic granules, which stain in an intense red after Diff-Quick. The role and clinical importance of this cell remain unclear, highlighting the need for further research into its characteristics and function.

#### 3.4.8 Reticulocytes

**Figure 10:**
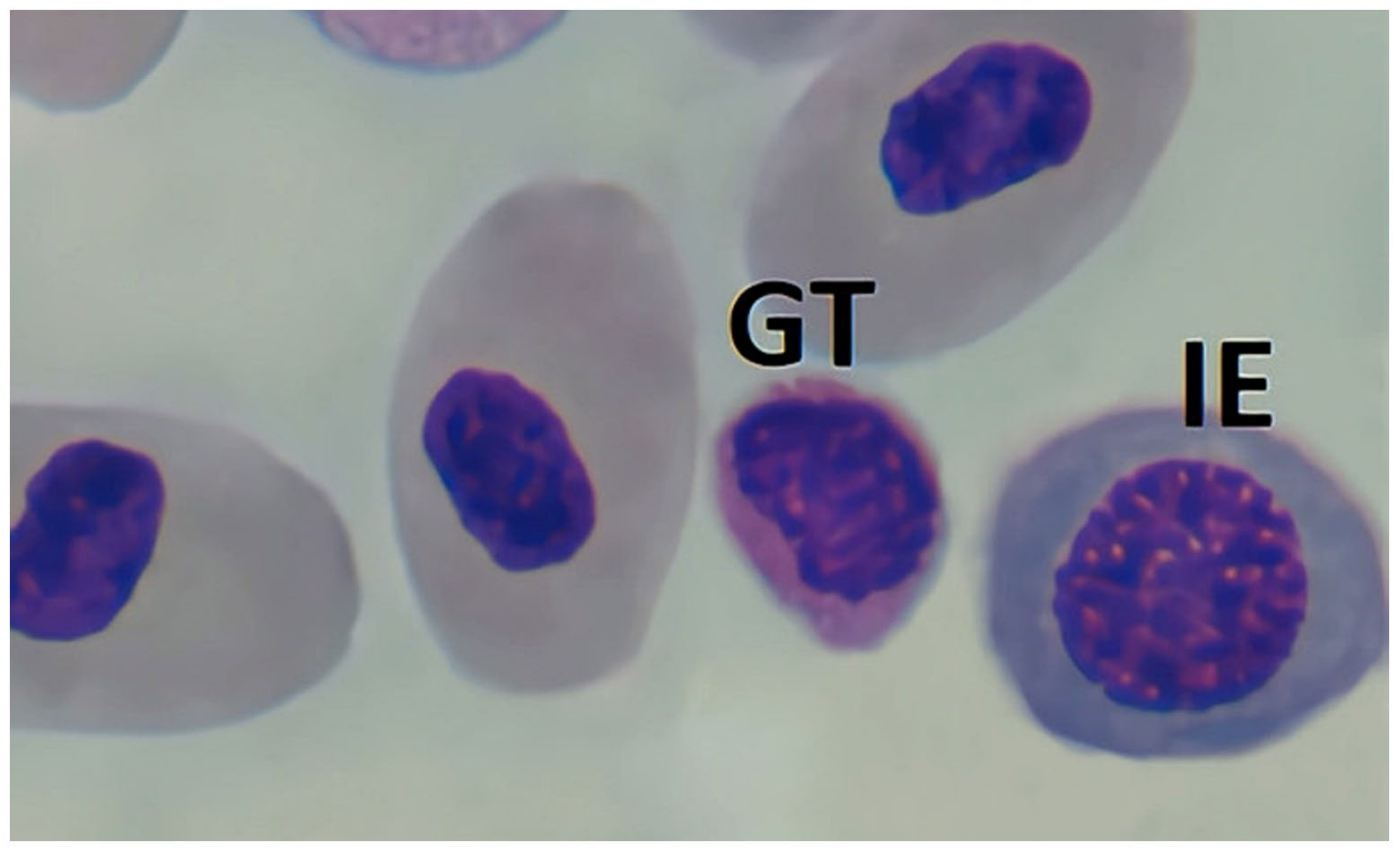
Blood smear of nursehound shark (*Scyliorhinus stellaris*) stained with Diff-quick, 100× magnification. A reticulocyte or immature erythrocyte (IE) can be seen as well as a granulated thrombocyte (GT).

As a last remark, care should be taken not to mistake reticulocytes, or immature erythrocytes, for lymphocytes. Some animals may present an abnormally high number of reticulocytes, special if anaemia is present, that would disrupt the leukocyte differentials if mistaken. These cells can be differentiated as they present a lower nucleus-to-cytoplasm ratio, and a clearly round nucleus (Figure 10).

### 3.5 Peripheral blood cells identification in a Neubauer improved chamber for total RBC, WBC and thrombocyte count, a visual guideline

**Figure 11:**
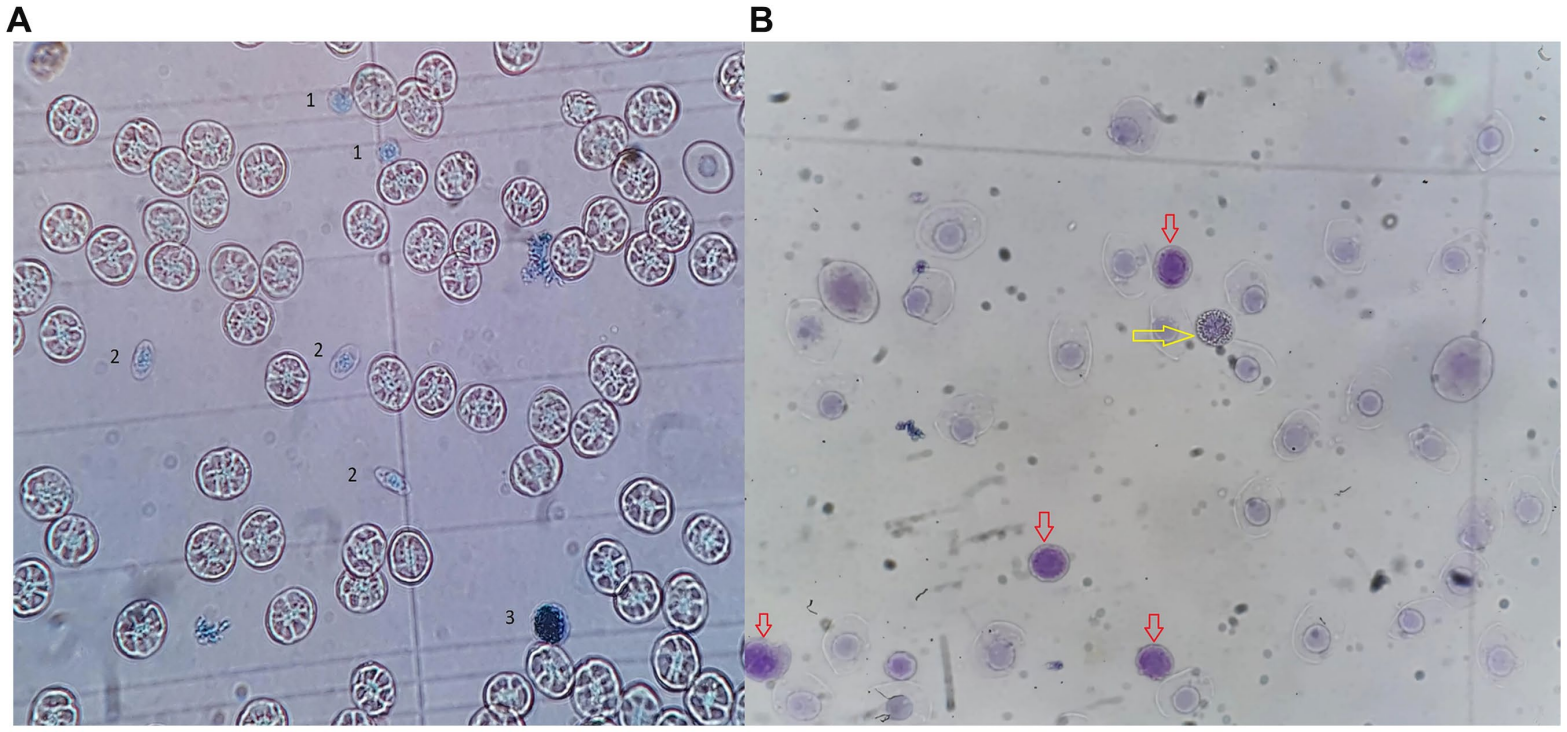
**(A)** Blood sample of an Atlantic devil ray (*Mobula hypostoma*) diluted in Rees-Ecker 1:100, loaded into Neubauer improved chamber, 100× magnification. Erythrocytes are the most present cells (E). 1: Lymphocytes; 2: Thrombocytes; 3: Granulocytes. **(B)** WBC count example in *Scyliorhinus stellaris*, Neubauer chamber, Natt Herricks note that the granulocyte (yellow arrow) can be differentiated from the immature RBC (red arrow) due to the granules.

Representative images of cell types are shown in Figure 11. Granulocytes are typically recognized as round, bright cells, while lymphocytes appear similar but are smaller in size. Care should be taken not to confuse early erythrocyte precursors (reticulocytes) with lymphocytes; reticulocytes generally exhibit a pale cytoplasm forming a halo around the nucleus and are less brightly stained. For further visual support on cell identification, both in blood smears and improved Neubauer chamber, please refer to the Supplementary figures.

### 3.6 Biochemistry

Blood chemistry analysis, including plasma and serum measurements, provides essential information about elasmobranch health (17, 18, 38). As this practice grows, reference values for diverse shark and ray species are being established, enhancing health assessments and comparative studies. However, research challenges, particularly limited access to large, uniform populations, typically result in studies with heterogeneous small sample sizes, which emphasizes the importance of expanded research with larger populations to enhance data interpretation.

Plasma obtained from centrifugation is preferred over serum and the sample should be used immediately after blood collection, as certain biochemical analytes like glucose can fluctuate over time. Consequently, reporting the time between collection and centrifugation is essential when presenting plasma biochemistry results. Appropriate centrifugation parameters must be established based on the required separation type: 450 g (approximately 2000 rpm, depending on the radius of the centrifuge) for 5 min. Marine-adapted elasmobranchs exhibit elevated sodium chloride and urea concentrations, as well as higher osmolality, potentially requiring serum or plasma sample dilution depending on the blood chemistry analyzer’s linear range. Given some laboratories’ limited experience with elasmobranch samples, critical analytical parameters may be overlooked when samples are sent to external facilities. Therefore, careful consideration of these species’ unique plasma biochemical characteristics is crucial when planning sample processing. The equipment used for biochemistry analysis should offer a wide detection range, as standard in-house biochemistry equipment often yields inadequate results. If in-house biochemistry is the only option, dilutions may be necessary at 1:10 for BUN, 1:4 for AST, LDH, and CK (David pers. com.). Ionograms (sodium, potassium, and chloride) cannot be diluted as this interferes with results and cannot be performed with such equipment. Systems capable of measuring electrolytes in urine samples should be sufficient for measuring electrolytes in elasmobranch serum or plasma. Additionally, clinicians should recognize that elasmobranchs regulate osmolality by absorbing Cl and Na in their intestines and excreting them via the rectal gland (39). They adapt to salinity changes by adjusting plasma osmolality, assisted by high levels of urea and TMAO. Urea increases osmotic pressure, while TMAO counteracts urea’s toxic effects (40, 41). Consequently, environmental factors such as salinity must be considered when interpreting certain analyte results in aquatic animals.

Research on individuals experiencing stressors such as capture, extended transport, or hypoxia from water removal has revealed elevations in intracellular potassium, magnesium, and ionized calcium levels, typically returning to baseline within 24 h after the stress event. Additionally, creatine kinase shows prolonged elevations, accompanied by significant increases in lactate, blood glucose, and plasma osmolality, alongside declining blood pH. Unlike most vertebrates where cortisol or corticosterone function as primary glucocorticoids, elasmobranchs predominantly produce 1α-hydroxy corticosterone, which increases during acute stress to facilitate energy mobilization. Despite evidence of glucose utilization as a metabolic substrate, circulating glucose levels generally remain relatively stable. In elasmobranchs, glucose levels remain consistent even during extended fasting. Previous studies have demonstrated that low glucose levels are characteristic of healthy individuals, whereas elevated glucose may indicate stress, such as during capture (42–46).

Contrary to what we see in other animals, where elevated levels of BUN may indicate kidney disease, among other changes, in elasmobranch the BUN value is linked to the nutritional condition of the animal, where lower values may infer a lower condition. Not every equipment reads BUN, some only read urea, so there may be the necessity to convert values through the following formula: BUN (mg/dL) = Urea (mg/dL)/2.1428. Authors have experienced that measuring Urea and converting to BUN may result in a different value from measuring BUN directly, which may be associated with the use of a different equipment for the same sample, so the method should be noted in every analysis performed (39, 40, 47, 48).

Creatine kinase levels vary considerably; in some cases, they fall below standard biochemical analyzers’ detection limits (<4 U/L) but can increase significantly depending on species and context. In cases of muscle catabolism, after prolonged and intense swimming, as well as repeated intramuscular injections, values may raise significantly. High levels are sometimes registered in routine examinations of apparently healthy animals. Similar interspecific and inter-study variability is observed in aspartate aminotransferase (AST) levels. Lactate dehydrogenase (LDH) also exhibits notable variation, often undetectable in certain species (7, 12, 18, 49, 50).

Alkaline phosphatase (ALP), which regulates phosphate absorption in the intestine and tissue mineralization similarly to other vertebrates like birds and mammals, demonstrates variability in elasmobranchs depending on diet, growth, and skeletal development. Diet variations, environmental factors (particularly salinity), age, developmental stage, and disease can also influence calcium and phosphorus levels. Gamma-glutamyl transferase (GGT), an enzyme found in hepatocyte membranes that maintains intracellular homeostasis and protects against oxidative stress, is typically very low in elasmobranchs, often below many biochemical analyzers’ detection limits. Nevertheless, GGT assessment remains important, as alterations in this enzyme have been associated with pathological conditions such as bile accumulation and liver injury, as well as age-related differences (51–54).

Significant variations in cholesterol, lipoproteins, ketone bodies, and triglyceride levels have been documented in elasmobranchs, frequently associated with seasonal changes and reproductive status. Previous studies have demonstrated a positive and statistically significant correlation between plasma triglyceride levels and body condition, suggesting that plasma triglyceride levels could serve as a reliable indicator of nutritional status, providing a valuable tool for health assessments and preventive medicine practices in these animals (17, 53, 54). Elasmobranchs’ energy metabolism is unique among vertebrates, as they lack fatty acid oxidation in skeletal and cardiac muscle, relying heavily on ketone bodies and amino acids as primary oxidative fuels in these tissues. This metabolic organization is reflected in low plasma levels of non-esterified fatty acids and elevated levels of ketone bodies, such as β-hydroxybutyrate, comparable to those observed in fasted mammals or teleost. Although ketone bodies are not currently frequently measured, including this analyte in blood analysis could provide valuable information on the animals’ general condition and metabolic status (46, 55).

Table 2 in the Results section summarizes key biochemical parameters analyzed across various elasmobranch species, revealing notable interspecies variability for most parameters.

**Table 2:** Compilation of the main biochemical parameters in different studied elasmobranch species.

Species (*n*)	*Rhinoptera bonasus* (18)	*Daasyais americana* (28)	*Carcharhinus melanopterus* (10)	*Cacharhinus taurus* (16)	*Rhina ancylostoma* (7)	*Aetobatus narinari* (15)	*Mustelus canis* (20)
ST	Me	Me	Me	Me	M	M	Me
ALP (U/L)	34.0	–	–	–	71.6	18.0	–
ALT (U/L)	BLD	–	–	–	3.2	–	–
AST (U/L)	33.0	14.5	47.0	22.0	21.5	8.47	11.3
GGT (U/L)	BDL	–	–	–	14.6	–	–
LDH (U/L)	BDL	BDL	382	–	102	–	BDL
PT (g/L)	28	26	34	27	32	–	30
BUN (mmol/L)	412	444	–	333	349	391	353
AU (μmol/L)	–	–	17.8	18.0	–	–	–
Col (mmol/L)	3.72	–	3.33	1.74	2.62	2.69	–
Trig (mmol/L)	1.77	–	–	0.37	–	–	–
CK (U/L)	BLD	80	1,455	233	545	99	7
Glu (mmol/L)	2.61	1.69	3.66	2.50	2.46	2.15	5.76
Ca (mmol/L)	4.2	4.1	4.0	3.7	3.7	4.1	4.2
Cl (mmol/L)	270	342	–	249.0	251.7	277.5	253.7
K (mmol/L)	1.5	4.9	3.6	4.0	4.24	4.47	4.1
Na (mmol/L)	294.0	315.0	280.0	257.0	242.5	287.5	255.3
P (mmol/L)	1.87	1.50	1.87	1.62	1.46	1.21	1.59
REF	(69)	(13)	(68)	(70)	(50)	(67)	(12)

Species (*n*)	*Rhincodon typus* (84)	*Rhizoprionodon terraenovae* (30)	*Sphyrna tiburo* (31)	*Squalus acanthias* (30)	*Carcharhinus limbatus* (36)	*Carcharhinus obscurus* (21)	*Carchachinus plumbeus* (12)
	Me	Me	Me	Me	M	Me	Me
ALP (U/L)	33	–	–	–	–	–	–
ALT (U/L)	8	6.5	BLD	13.0	–	–	–
AST (U/L)	10	25.5	39.0	7.0	6	51	51
GGT (U/L)	1	–	–	–	5	28	17
LDH (U/L)	53.8	–	–	–	–	–	–
PT (g/L)	15	23	28	21	22	4.2	3.9
BUN (mmol/L)	881	–	–	–	–	1,111	2,390
AU (μmol/L)	11.9	35.7	47.6	BLD	–	–	–
Col (mmol/L)	0.71	2.22	2.82	2.53	1.16	1.91	2.84
Trig (mmol/L)	0.11	BDL	0.32	0.34	0.46	0.53	0.49
CK (U/L)	58.5	258	172	BDL	23	4,589	7,393
Glu (mmol/L)	47	9.27	9.71	2.33	58	84	65
Ca (mmol/L)	14.4	4.6	4.7	3.4	16	15.4	15.8
Cl (mmol/L)	228	–	–	–	277.4	302	295
K (mmol/L)	5	5.9	6.0	4.0	3.35	4.6	4
Na (mmol/L)	279	–	–	–	287	292	282
P (mmol/L)	1.9	2.31	2.35	1.42	–	5.6	5.9
REF	(21)	(7)	(7)	(7)	(66)	(22)	(22)

ST, statistic; Me, median; M, arithmetic mean; ADL, values above the detection limit; BDL, values below the detection limit; ALP, alkaline phosphatase; ALT, alanine aminotransferase; AST, aspartate aminotransferase; GGT, gamma-glutamyl transferase; LDH, lactate dehydrogenase; PT, total plasma proteins; BUN, blood urea nitrogen; AU, uric acid; Col, total cholesterol; Trig, triglycerides; CK, creatine kinase; Glu, glucose; Ca, calcium; Cl, chloride; K, potassium; Na, sodium; P, phosphorus; REF, reference.

In mammals and reptiles, a rise in phosphorus levels or a decrease in the calcium/phosphorus ratio are linked with kidney failure. Even though this has not been established in elasmobranchs, any significant changes in these parameters would warrant a thorough complementary diagnostic evaluation, as the author has seen a ray with urinary calculus exhibit a marked rise in phosphorus levels (56, 57).

### 3.7 Plasma protein electrophoresis

Although the number of studies defining reference intervals for hematological and plasma biochemical parameters in elasmobranchs is steadily growing, plasma protein electrophoresis remains underrepresented, with guideline values established for only a limited number of species. This likely reflects the restricted access to electrophoresis equipment in many facilities, as well as a general lack of awareness among clinicians regarding the diagnostic value of this tool, particularly in non-domestic species.

Electrophoretic analyses in sharks and rays have revealed five to seven consistent protein fractions, typically including pre-albumin, albumin, alpha (often subdivided into alpha 1 and alpha 2), beta (sometimes divided into beta 1 and beta 2), and gamma globulins. However, the presence and clinical significance of albumin in elasmobranchs remains a topic of debate. Several studies have documented extremely low or even undetectable levels of albumin in species such as the bamboo shark, bonnethead shark, and cownose ray (23, 24, 58–60). One investigation involving eight chondrichthyan species reported no measurable albumin at all, suggesting that these animals may not produce meaningful quantities of unesterified fatty acids, instead relying on protein binding of long-chain fatty acids for lipid transport (61). This tool has also been shown to detect subclinical disease, monitor protein trends over time, and provide accurate lipoprotein quantification, valuable in evaluating inflammation and immune responses, all without the need for species-specific reagents (35, 60, 62). Table 3 in Results summarizes plasma protein electrophoresis results for multiple elasmobranch species, highlighting considerable interspecific variation in both fraction profiles and absolute concentrations.

**Table 3:** Compilation of plasma protein electrophoresis results published for different elasmobranch species.

Species (*n*)	*Rhinoptera bonasus* (35)	*Cacharhinus taurus* (20)	*Chiloscyllium plagiosum* (20)	*Sphyrna tiburo* (23)	*Sphyrna tiburo* (31)	*Rhizoprionodon terraenovae* (30)	*Squalus acanthias* (30)
ST	Me	Me	Me	Me	Me	Me	Me
TP (g/L)	54	33	70	62	28	23	21
A/G	0.03	0.1	0.02	0.05	0.05	0.14	0.2
F1 (%)	5.60	0.0	0.0	4.82	0.71	1.30	6.67
F2 (%)	2.90	1.33	1.86	4.37	3.57	10.87	9.52
F3 (%)	12.90	2.00	1.42	36.02	2.50	3.91	2.38
F4 (%)	68.40	62.67	21.71	47.82	46.78	44.00	68.57
F5 (%)	8.90	8.00	59.86	6.38	3.57	4.35	4.76
F1 (g/L)	2.9	0.3	0.0	3.2	0.2	0.3	1.4
F2 (g/L)	1.6	0.6	1.3	2.8	1.0	2.5	2.0
F3 (g/L)	7.2	8.7	1.0	22.3	0.7	0.9	0.5
F4 (g/L)	38.3	19.6	41.9	29.4	13.1	12.0	14.4
F5 (g/L)	4.8	2.7	8.5	4.1	1.0	1.0	1.0
REF	(35)	(70)	(71)	(23)	(7)	(7)	(7)

ST, statistical method; Me, median; *n*, number of individuals; SD, standard deviation; TP, total proteins; A/G ratio, Albumin/Globulin ratio or “fraction 1: fractions 2–5 ratio”; F1, fraction 1 – equivalent to prealbumin; F2, fraction 2 – equivalent to albumin; F3, fraction 3 – equivalent to alpha-globulins; F4, fraction 4 – equivalent to beta-globulins; F5, fraction 5 – equivalent to gamma-globulins. Please note: in some cases, total plasma solids were measured instead of total proteins, with fractions calculated from these values. Fraction 3 is divided into two subfractions in certain species, corresponding to alpha-1 and alpha-2 globulins.

## 4 Results

The consistent application of this standardized protocol will yield a comprehensive and reliable hematological and biochemical profile for elasmobranchs, enhancing both clinical diagnostics and research data. The expected outcomes, along with their inherent advantages, limitations, and troubleshooting measures, are detailed below.

Upon successful execution, the protocol will generate a suite of data including blood gas values including pH, pO2, pCO₂, HCO3- and lactate; a complete blood count which comprises PCV, RBC, WBC, thrombocytes, and differential leukocyte count; plasma biochemistry; a plasma protein electrophoretogram. A primary advantage is the direct comparability of results across different individuals, institutions, and studies, which has previously been a significant challenge (8, 17). The hematological analysis will reliably identify the distinct leukocyte populations characteristic of elasmobranchs, such as fine and coarse eosinophilic granulocytes, and in some species, granulated thrombocytes, as illustrated in the cell identification guide (Methods). The expected values for these parameters across various species are compiled in Table 1, demonstrating the considerable interspecific variability that underscores the need for species-specific references. For biochemistry, the protocol is designed to accurately measure the uniquely high levels of urea and electrolytes in marine elasmobranchs, with expected ranges provided in Table 2. A key outcome of the electrophoresis component is the consistent separation of five to seven protein fractions (Table 3), with CZE offering superior resolution over traditional agarose gels to better differentiate these fractions, which is crucial for detecting inflammatory or pathological shifts (62, 63).

Several limitations are inherent to elasmobranch blood analysis. A significant limitation of in-house biochemistry analyzers is their often-narrow linear range, which can lead to values being “above detection limit” for enzymes like CK and AST after muscle exertion, or “below detection limit” for GGT (17). In hematology, the use of a standard refractometer for plasma protein estimation is unreliable due to interference from high urea and electrolyte concentrations (24, 35). Common pitfalls include the misidentification of cells on blood smears; for example, reticulocytes can be mistaken for lymphocytes, and granulocytes with overlapped nuclear segmentation may be incorrectly classified as immature, potentially leading to a misdiagnosis of a “left shift” (11). Pre-analytical artifacts are a major concern. Stress from prolonged restraint can induce metabolic acidosis, elevating lactate and depressing pH (28, 29). The use of sodium heparin as an anticoagulant can artifactually increase measured sodium levels, and delays in processing can cause *in vitro* changes in blood gas values and glucose concentration.

To counteract these issues, a rigorous troubleshooting approach is required. To address analytical limitations, plasma samples for biochemistry should be diluted (e.g., 1:10 for BUN) when using standard equipment, and results should always be interpreted with knowledge of the analyzer’s detection limits. For accurate protein quantification, biochemical methods are strongly preferred over refractometry (36). To prevent cell misidentification, technicians should be trained using a standardized guide and should always examine smears in the monolayer region to avoid distorted cells. Differentiating lymphocytes from thrombocytes is based on cytoplasmic colour and shape, while reticulocytes are identified by their lower nucleus-to-cytoplasm ratio and pale, halo-like cytoplasm. To minimize pre-analytical artifacts, handling time must be minimized, and comprehensive metadata must be recorded, including restraint duration, venipuncture site, and time to centrifugation. Blood gas analysis should be performed immediately from a lightly heparinized syringe. If electrolyte analysis is required, a separate sample should be drawn into a lithium heparin tube. By adhering to these troubleshooting measures, clinicians and researchers can ensure the generation of high-quality, interpretable data that faithfully reflects the animal’s physiological state.

## 5 Discussion

The standardization of blood collection and analytical protocols in elasmobranchs represents a critical advancement toward maximizing the diagnostic value and cross-study comparability of hematological assessments in these remarkable species. Despite significant progress and growing interest in elasmobranch medicine, the field continues to navigate substantial challenges related to methodological consistency, comprehensive reference intervals, and species-specific data interpretation frameworks (2, 17). The validation of these methods is evidenced by their successful application in generating the reference data compiled within this work and their foundation in both published literature and extensive clinical practice. This guideline serves as a comprehensive compilation of evidence-based practices for blood sampling techniques, blood gas analysis, hematological evaluation, biochemical profiling, and plasma protein electrophoresis. It synthesizes contemporary scientific literature with practical, field-validated methodologies developed through extensive clinical experience. The protocols outlined herein have been deliberately designed to be implementable using equipment and materials typically accessible in veterinary clinical settings, public aquariums, zoological institutions, and marine research facilities, thus ensuring their practical utility across diverse contexts.

The step-by-step procedures outlined for venipuncture, blood gas analysis, hematology, biochemistry, and plasma protein electrophoresis are designed to be implementable with common veterinary equipment, thereby maximizing their practical utility. A key advantage of this harmonized approach is its potential to support the development of robust, longitudinal databases, which are indispensable for establishing statistically valid, species-specific reference intervals, a foundational element currently lacking in elasmobranch medicine (22). For instance, the consistent use of modified Natt-Herrick’s solution and the formalin-based preservation technique for delayed cell counts address specific analytical pitfalls related to elasmobranch blood osmolarity and sample stability (2, 37).

The anticipated results of widespread protocol adoption are multifaceted. In clinical settings, it enables earlier detection of pathologies and more precise monitoring of treatment efficacy, as demonstrated by the ability of electrophoresis to identify subclinical disease and inflammatory profiles (23). Beyond immediate clinical applications, the widespread adoption of harmonized blood analysis protocols across the global community of elasmobranch-holding institutions will significantly elevate the standard of veterinary care available to these animals. In research, it allows for more meaningful meta-analyses and a better understanding of physiological responses to environmental and anthropogenic stressors (20, 45). Furthermore, it will contribute substantially to advancing our scientific understanding of elasmobranch health dynamics, disease processes, and conservation requirements/knowledge that has implications not only for captive animal welfare but also for wild population management and conservation initiatives.

However, the limitations of these methods must be acknowledged. The precision of biochemical analysis is heavily dependent on the analytical equipment’s detection range, often requiring sample dilution for analytes like urea or yielding values below detectable limits for enzymes like GGT and CK in some species (18, 33). Potential artifacts can arise from improper handling, such as the continued *in vitro* gas exchange, due to plastic syringes gas permeability, that alter blood gas values if analysis is delayed (64). Additionally, since fish erythrocytes are nucleated, they perform aerobic respiration and they will remain metabolically active after the blood is collected into the syringes, which causes reduction in pO2 and pH, and increases pCO2 (65). Considering all this information, if samples are collected for blood gas analysis and are not readily applied into the chemistry analyzer, they should be stored in glass gas-tight 1 mL syringes that have been packed in ice before, and after sampling, the syringes should be immediately repacked in ice until blood samples are injected into the cartridges. Another possible artifact is the misclassification of immature granulocytes and reticulocytes on blood smears if smear quality is poor or examination is not meticulous (11, 26). Troubleshooting these issues requires strict adherence to timing, the recording of comprehensive metadata (e.g., handling time, equipment used), and the development of technician proficiency through repeated exposure to species-specific cellular morphology.

The broad implementation of standardized protocols for blood collection and analysis among institutions maintaining elasmobranchs, as well as professionals working with these taxa, will substantially enhance the quality and consistency of veterinary diagnostics and clinical management (1, 9, 10). Moreover, methodological harmonization will generate more comparable and robust datasets, thereby advancing our understanding of elasmobranch physiology, health status, and disease processes. Such improvements in data quality have direct relevance not only for animals under professional care but also for the assessment, monitoring, and conservation of free-ranging populations, ultimately supporting evidence-based management strategies at both local and global scales (20, 66).

As our understanding of elasmobranch blood analysis continues to evolve, regular refinement of these guidelines will ensure they remain aligned with emerging research and technological developments (4, 5). Through collaborative implementation and ongoing improvement of these standardized approaches, the veterinary and research communities can collectively enhance both the welfare of individual animals and the broader body of knowledge supporting elasmobranch medicine, research and conservation.

## Data Availability

The original contributions presented in the study are included in the article/Supplementary material, further inquiries can be directed to the corresponding authors.
