# Supplementary material for: Standardized protocols for blood collection and analysis in elasmobranchs: a practical guide for clinicians and researchers
Source: Front Vet Sci. 2026 Jan 20;12:1754037. doi: 10.3389/fvets.2025.1754037 (PMC12866614; doi:10.3389/fvets.2025.1754037)
Supplement: Supplementary file 1 [file Table_1.DOCX]

Supplementary Material

# Main venipuncture sites used in elasmobranchs

**Supplementary Figure 1**. Parasagittal cut of an Atlantic devil ray (*Mobula hypostoma*) wing, with the ventral surface seen on the upper side. Blood vessels (yellow arrows) can be seen between the cartilage segments.

**Supplementary Figure 2**. Sagittal cut of a zebra shark (Stegostoma tigrinum) tail. Left of the image is dorsal and right is ventral. The needle placement shows the best approach for the caudal vein in this species. The caudal vein (yellow arrow) is fully covered by cartilage.

**Supplementary Figure 3**. Caudal cross-section in a nursehound (*Scyliorhinus stellaris*), with dorsal at the top and ventral at the bottom. Note that when using the lateral approach to access the vessel for venipuncture, the amount of muscle tissue to be penetrated is less than with the ventral approach. This varies between species.

**Supplementary Figure 4.** Blood collection in a zebra shark (*Stegostoma tigrinum*) showing the lateral approach to the caudal vein.

# Visual guide to leukocyte identification in stained blood smears

Supplementary Figure 5. Macroscopic picture of a blood smear indicating appropriate area for differential leukocyte counts (yellow rectangle).

**Supplementary Figure 6.** A 100x magnification of a blood smear, stained with Diff-Quick indicating appropriate cell density for differential leukocyte counting.

**Supplementary Figure 7.** Blood smear of zebra shark (*Stegostoma tigrinum*), stained with Diff-quick, 100x magnification. Here we can appreciate the differences between a Neutrophil (N), a Fine eosinophilic granulocyte (FEG), and a coarse eosinophilic granulocyte (CEG). Lymphocytes (L) and thrombocytes (T) can also be seen.

**Supplementary Figure 8.** Blood smear of a giant guitarfish (*Glaucostegus cemiculus*), stained with Diff-quick, 100x magnification. Here we can appreciate the differences between: (A) a Fine eosinophilic granulocyte (FEG) ; (B) a coarse eosinophilic granulocyte (CEG).

**Supplementary Figure 9.** Blood smear of zebra shark (*Stegostoma tigrinum*), stained with Diff-quick, 100x magnification. A monocyte (M) can be seen.

**Supplementary Figure 10.** Blood smear of a Nursehound shark (*Scyliorhinus stellaris*), stained with Diff-quick, 100x magnification. Thrombocytes (T), granulated thrombocytes (GT), a neutrophile (N) and a coarse eosinophilic granulocyte (CEG) can be seen. Please note the different staining properties between the GT and the T.

**Supplementary Figure 11.** Blood smears of a Nursehound shark (*Scyliorhinus stellaris*), stained with Diff-quick, 100x magnification, differentiating thrombocytes (T) from granulated thrombocytes (GT).

**Supplementary Figure 12.** Blood smear of zebra shark (*Stegostoma tigrinum*), stained with Diff-quick, 100x magnification. A reticulocyte or immature erythrocyte (IE) can be seen, as well as a neutrophil (N), two lymphocytes (L) and a thrombocyte (T).

**Supplementary Figure 13.** Blood smears of Atlantic devil rays (*Mobula hypostoma*) stained with Diff-Quick, 1000x magnification. A – Fine eosinophilic granulocyte (arrow), thrombocyte (yellow arrowhead) and lymphocyte (blue arrow); B – Coarse eosinophilic granulocyte (arrow) and thrombocytes (yellow arrowheads); C – Basophil (black arrow), thrombocyte (yellow arrowheads) and lymphocyte (blue arrow); D – Monocyte (black arrow)

# Visual guide to cell identification in improved Neubauer chamber

**Supplementary Figure 14.** Blood sample of a zebra shark (*Stegostoma tigrinum*) diluted in Rees-Ecker 1:100, loaded into Neubauer improved chamber, 100x magnification. Reticulocyte (yellow arrow).
