# Supplementary figures and images for: Standardized protocols for blood collection and analysis in elasmobranchs: a practical guide for clinicians and researchers
Source: Front Vet Sci. 2026 Jan 20;12:1754037. doi: 10.3389/fvets.2025.1754037 (PMC12866614; doi:10.3389/fvets.2025.1754037)

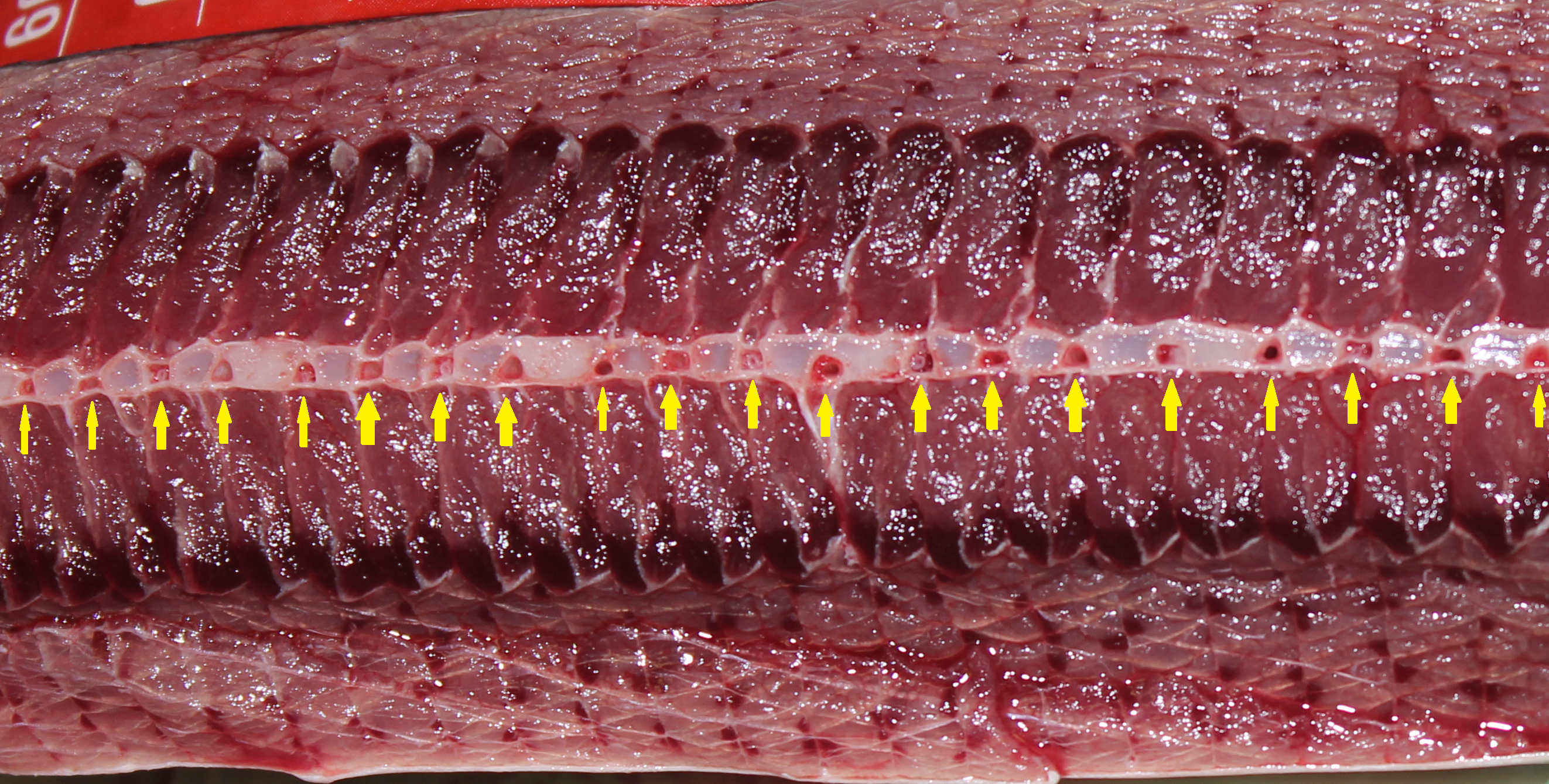

Supplement: Supplementary file 2 [file Image_1.tif]

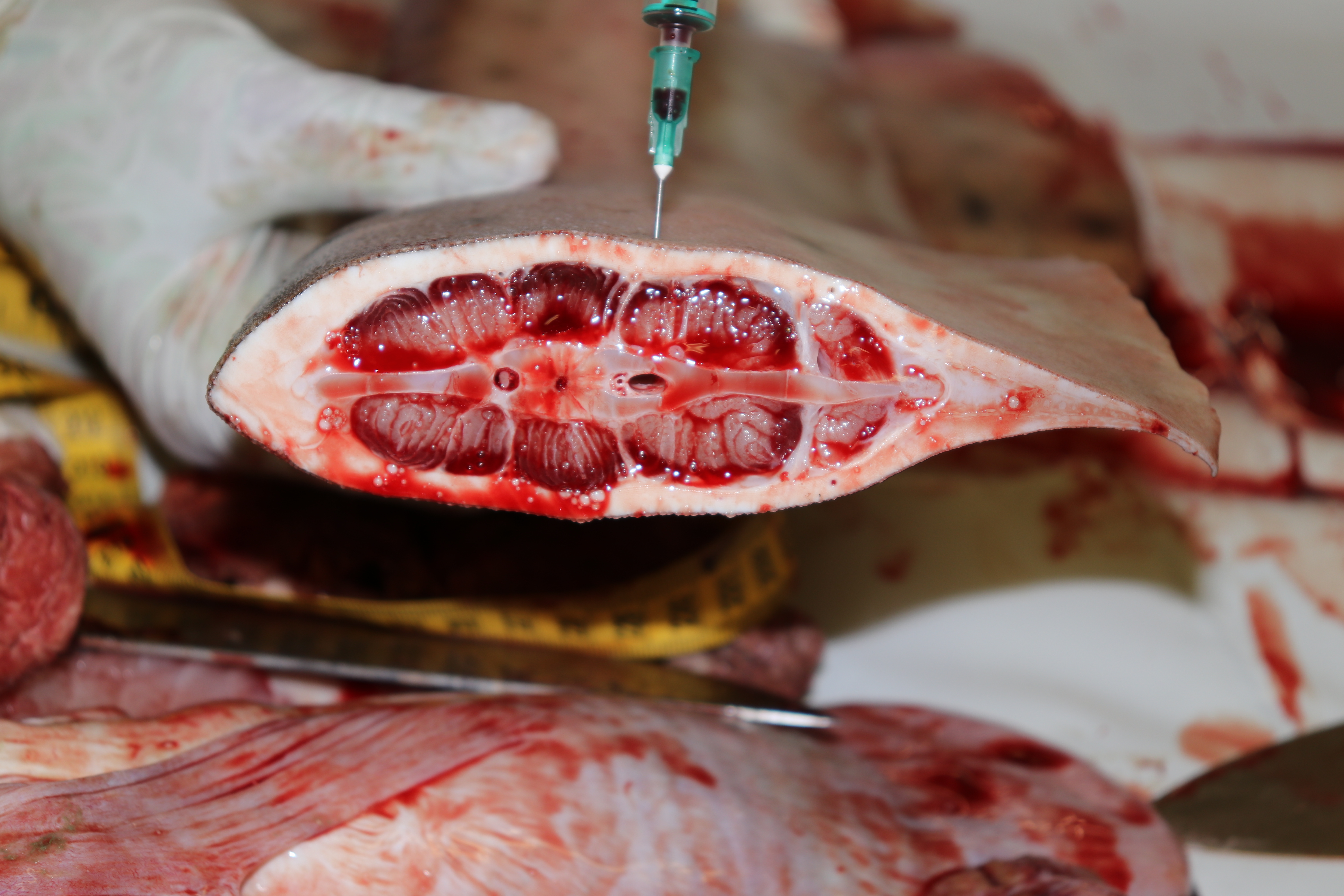

Supplement: Supplementary file 3 [file Image_2.jpeg]

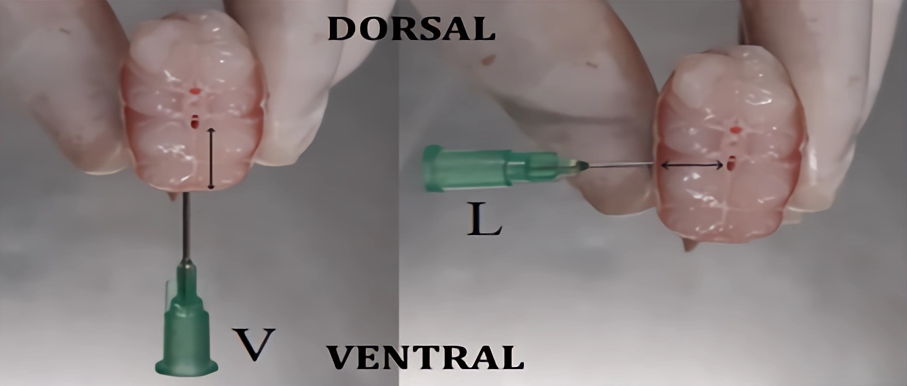

Supplement: Supplementary file 4 [file Image_3.tif]

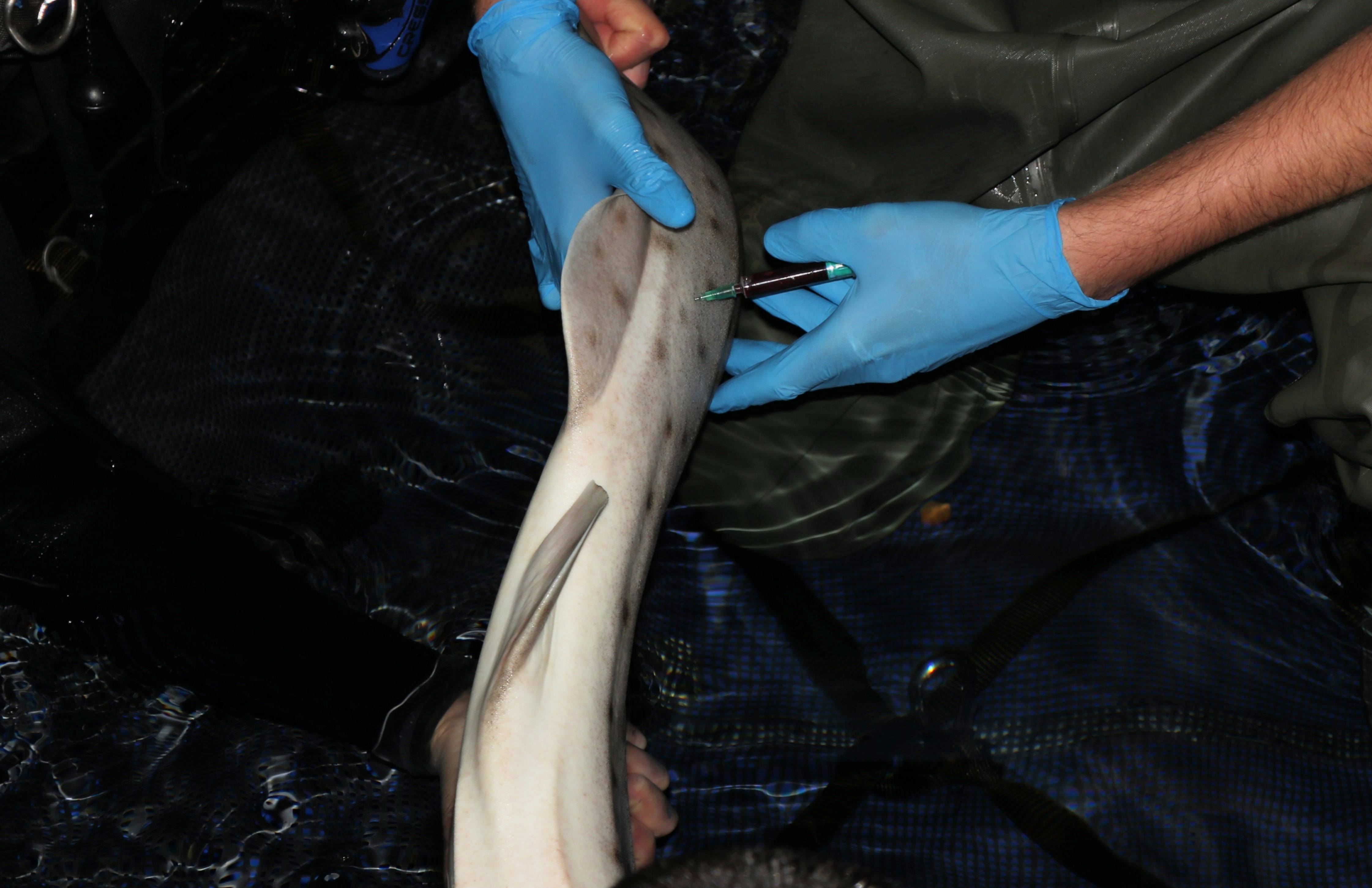

Supplement: Supplementary file 5 [file Image_4.jpeg]

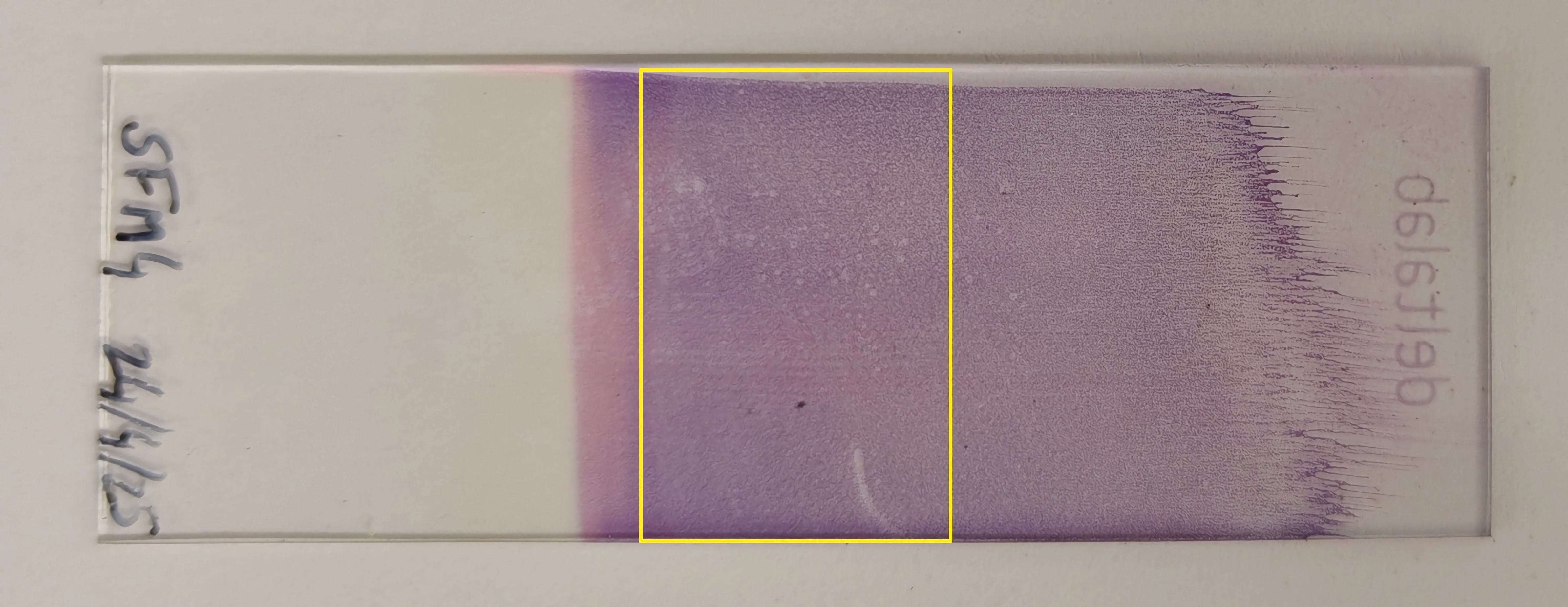

Supplement: Supplementary file 6 [file Image_5.jpeg]

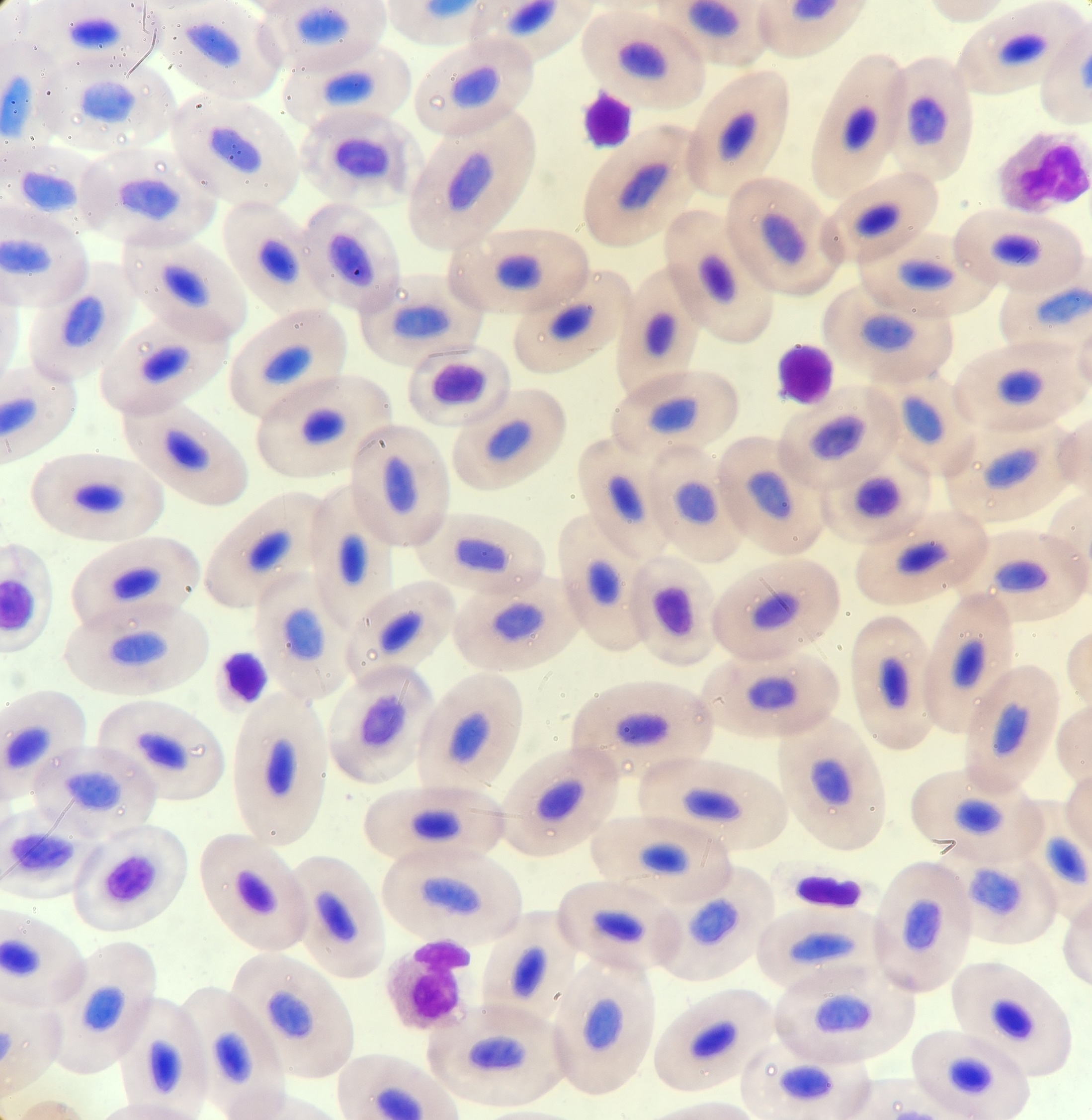

Supplement: Supplementary file 7 [file Image_6.jpeg]

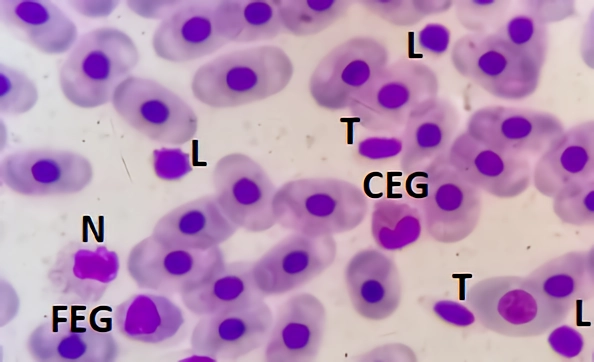

Supplement: Supplementary file 8 [file Image_7.tif]

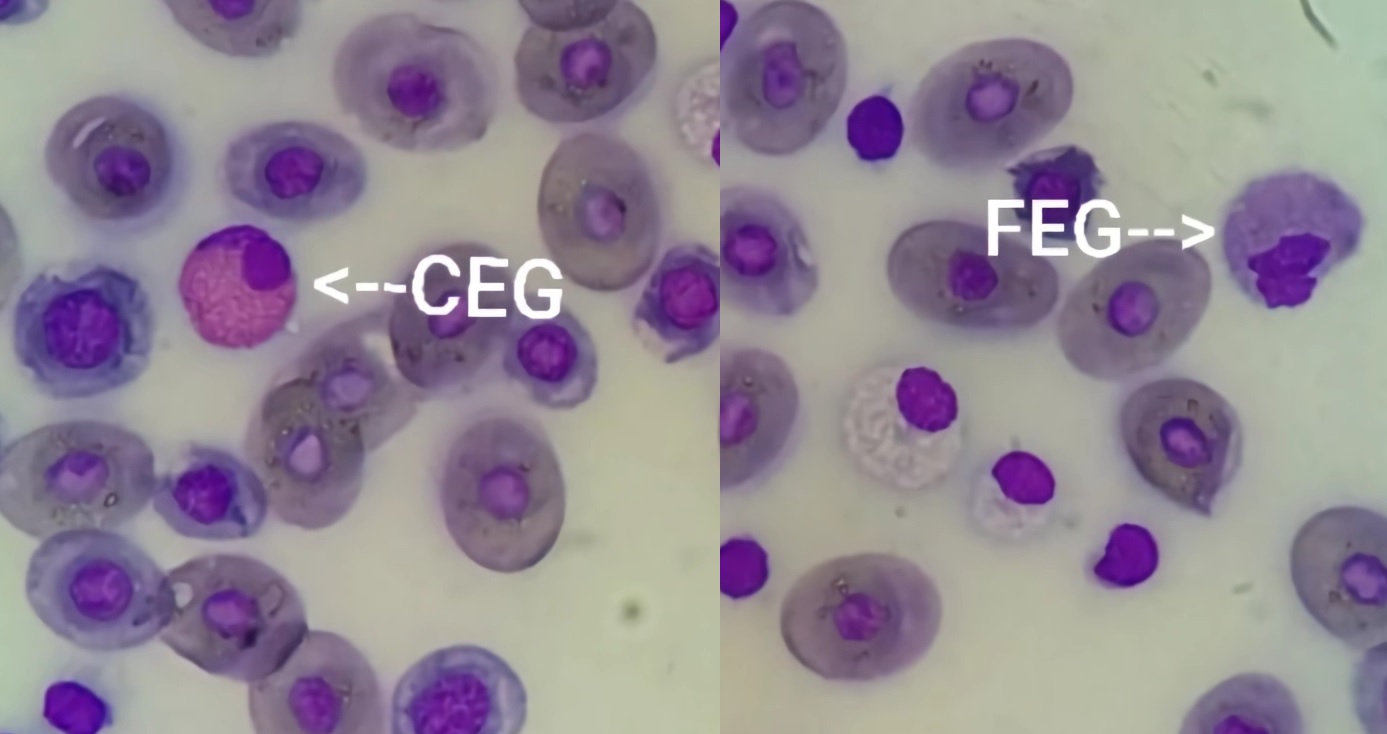

Supplement: Supplementary file 9 [file Image_8.jpeg]

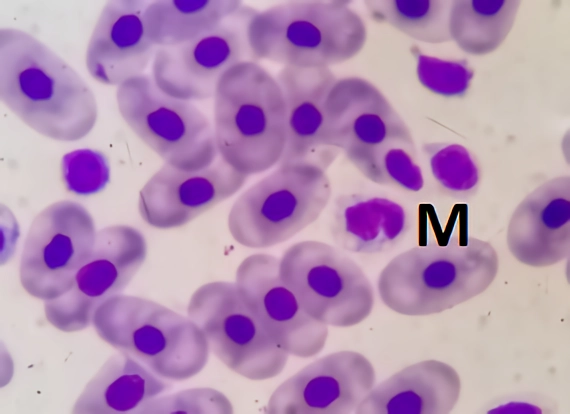

Supplement: Supplementary file 10 [file Image_9.tif]

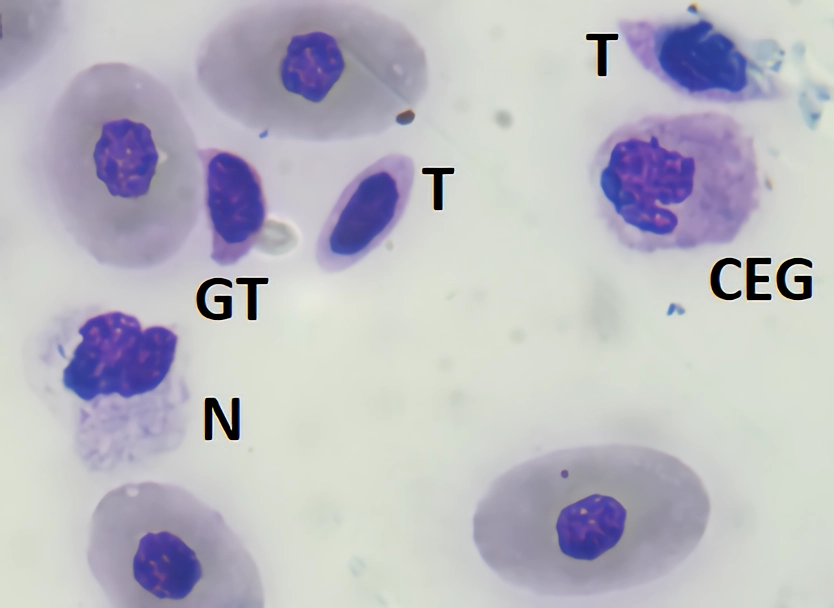

Supplement: Supplementary file 11 [file Image_10.tif]

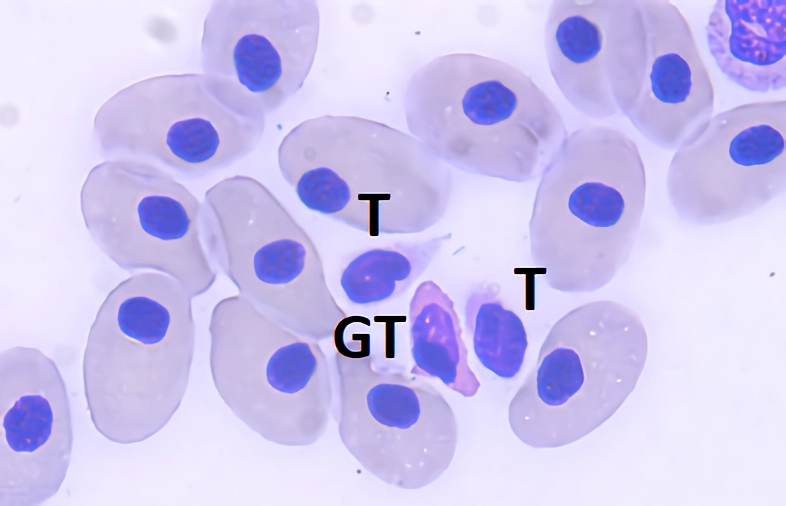

Supplement: Supplementary file 12 [file Image_11.tif]

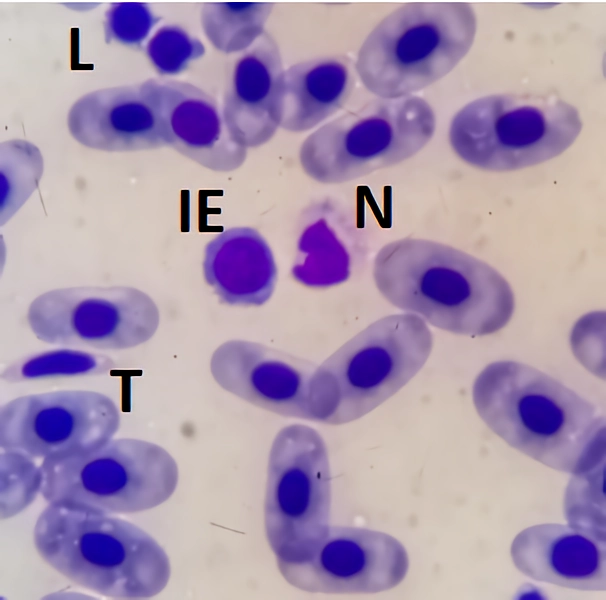

Supplement: Supplementary file 13 [file Image_12.tif]

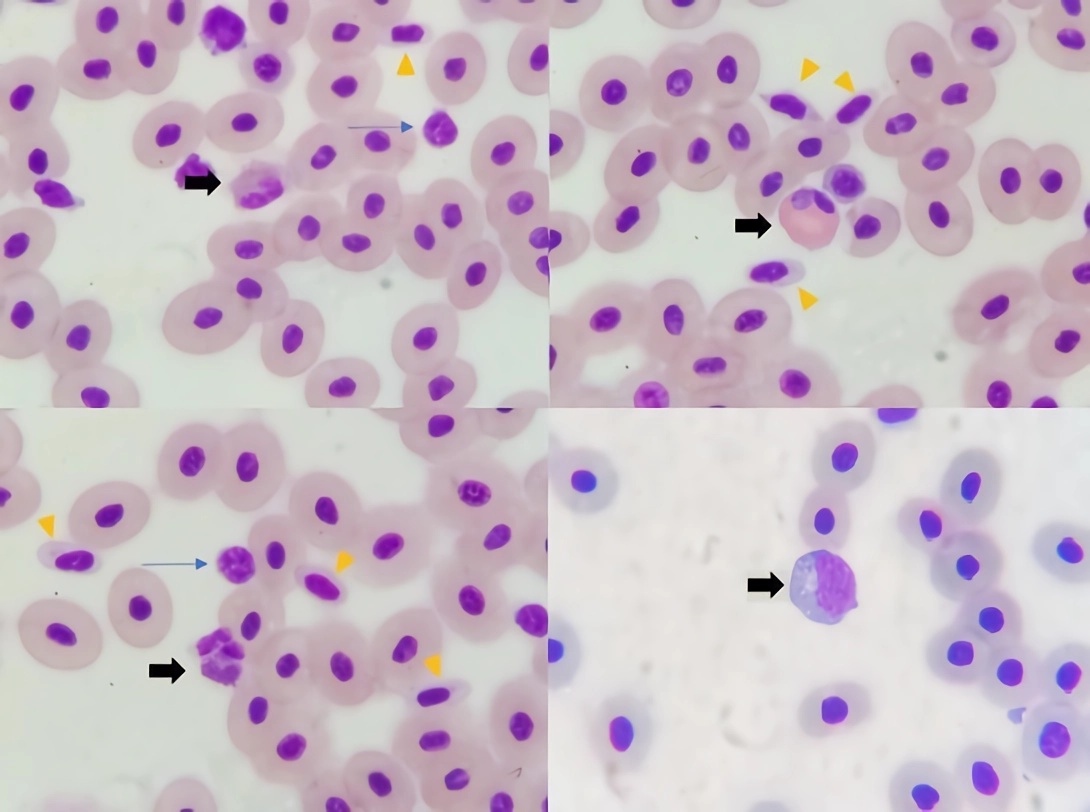

Supplement: Supplementary file 14 [file Image_13.jpeg]

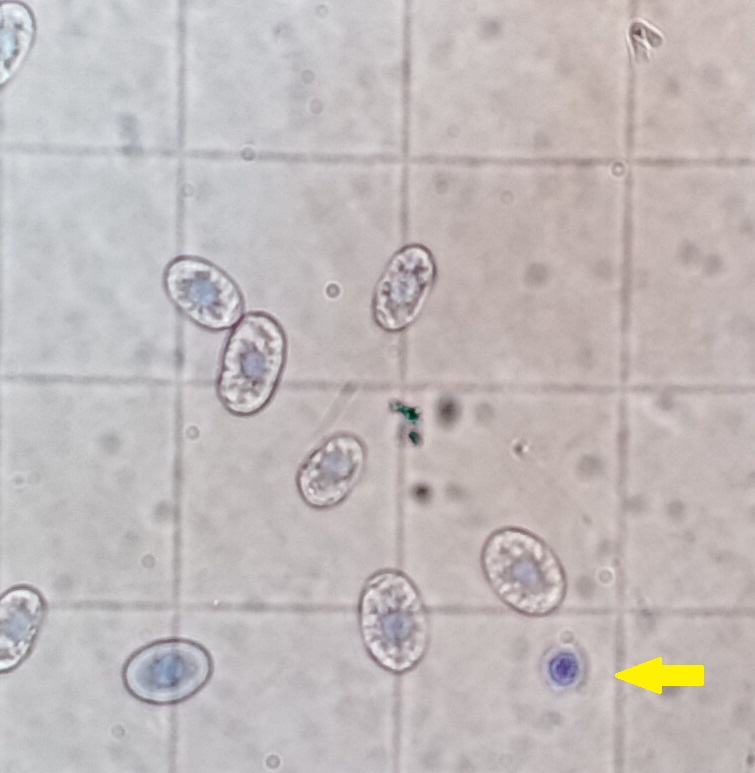

Supplement: Supplementary file 15 [file Image_14.jpeg]
